# Effects of Mechanical Stress on Endothelial Cells In Situ and In Vitro

**DOI:** 10.3390/ijms242216518

**Published:** 2023-11-20

**Authors:** Kazuo Katoh

**Affiliations:** Laboratory of Human Anatomy and Cell Biology, Faculty of Health Sciences, Tsukuba University of Technology, Tsukuba 305-8521, Japan; katoichi@k.tsukuba-tech.ac.jp

**Keywords:** mechanical stress, endothelial cells, blood vessel, signal transduction, stress fiber, focal adhesion, cytoskeleton

## Abstract

Endothelial cells lining blood vessels are essential for maintaining vascular homeostasis and mediate several pathological and physiological processes. Mechanical stresses generated by blood flow and other biomechanical factors significantly affect endothelial cell activity. Here, we review how mechanical stresses, both in situ and in vitro, affect endothelial cells. We review the basic principles underlying the cellular response to mechanical stresses. We also consider the implications of these findings for understanding the mechanisms of mechanotransducer and mechano-signal transduction systems by cytoskeletal components.

## 1. Introduction

Blood flow and tissue deformation continuously subject endothelial cells, which form the inner lining of arteries, to mechanical stresses [1]. Hydrostatic pressure, shear stress, and cyclic strain are some of the mechanical factors important in maintaining vascular homeostasis and modulating endothelial cell activity [2,3,4]. Mechanical stresses affect processes such as gene expression, cell shape, migration, proliferation, and the release of various bioactive substances. Under various physiological and pathological conditions, these responses are tightly regulated and are used to determine whether changes in endothelial function are adaptive or maladaptive [5]. Understanding the effects of mechanical stresses on endothelial cells is essential for understanding the intricate mechanisms behind vascular physiology and the pathogenesis of vascular diseases. Vascular health depends on the integrity and optimal function of the endothelium. According to Sandro et al. [6], the endothelium serves as a dynamic interface between the blood and the underlying tissues.

The methods by which mechanical stresses are sensed in the endothelium remain largely unknown. However, ion channels and integrins have been suggested as possible sensors. The mechanotransduction molecule platelet endothelial cell adhesion molecule-1 (PECAM-1) has also been discovered [7,8,9,10]. The specific location of these sensors is unknown. However, they may be located on the apical surface or transduced via cytoskeletal structures such as actin filaments (actomyosin-based contractile systems) and focal adhesions (cell-substrate adhesion sites) [11]. In addition, apical plaques and cell–cell contacts may be important for sensing and signaling [12,13].

Our understanding of the effects of mechanical stresses on endothelial cells has benefited greatly from in situ research using intact arteries from living organisms and from in vitro experiments using cultured endothelial cells. In situ studies reveal the complex relationships between mechanical stressors, vascular physiology, and endothelial function in the context of the entire vascular system. In vitro studies provide a platform to study specific cellular processes and communication pathways while allowing for controlled adaptation of mechanical stimuli [14].

Endothelial cells are exposed to mechanical stress outside the range of normal physiology, which has a major impact on many disease processes. The development of aneurysms, diabetic vasculopathy, atherosclerosis, and hypertension are examples of vascular diseases that share the characteristic of a dysfunctional endothelium. According to several studies, mechanical stress affects endothelial cell activity, increases endothelial dysfunction, and initiates pro-atherogenic, pro-inflammatory, and pro-thrombotic responses that influence the onset, course, and outcome of many diseases [2,3,15].

In order to treat vascular disease and restore endothelial function, innovative treatment techniques can be developed that specifically target mechanotransduction pathways [4,16]. This review examines the effects of mechanical stress on endothelial cells, in situ and in vitro, for normal and pathological systems.

## 2. In Situ Studies of Endothelial Cell Response in Blood Vessels

### 2.1. Hemodynamic Forces of Blood Vessels

#### 2.1.1. Wall Shear Stress

The force exerted by the movement of blood against the arterial wall is known as wall shear stress (WSS). This stress is thought to play a critical role in the adaptation of the vascular wall by triggering the production of substances such as endothelin, prostacyclin, and nitric oxide in endothelial cells [17]. Shear stress is defined as the pressure applied to the surface of the vessel per unit area [18,19]. The evaluation of shear stresses acting on a surface is based on a fundamental principle of fluid mechanics, which assumes that the fluid velocity at the surface is zero (known as the no-slip condition). This condition results in the creation of a velocity gradient. Consequently, as fluid particles move parallel to the surface, their velocity increases from zero near the surface to a maximum value at a certain distance from the surface [20]. The term “shear rate” refers to the rate at which adjacent fluid layers undergo relative motion. The shear rate is proportional to the shear stress in the vessel. The wall shear rate in a healthy artery without blood clots is usually about 1000 s^−1^, but in clogged arteries, it can range from 5000 to 400,000 s^−1^.

A blood vessel can be thought of as a linear and cylindrical conduit. If the walls of the vessel are rigid, the velocity gradient (shear rate), denoted by ỳ, can be determined using the following relationship:

(1) $$ỳ=\frac{du}{dr}$$

In this context, the symbol “*u*” represents the fluid velocity and “*r*” represents the radius of the vessel. Here, we discuss two different situations involving fluid flow with different velocity profiles and velocity gradients. Endothelial cells in macrovessels are exposed to mechanical forces such as shear stress that are significantly different from those in microvessels, which are characterized by low shear stress and different hemodynamic flow patterns. The fluid is blood, which we approximate as an ideal Newtonian fluid of constant viscosity. The assumption of Newtonian behavior in modeling blood flow is a simplification. Blood is a complex fluid that can exhibit non-Newtonian behavior, such as shear thinning, under certain conditions. However, in many cases, assuming Newtonian behavior simplifies calculations and provides reasonable approximations for flow in larger blood vessels [21,22]. The flow is assumed to be steady and laminar, while the vessel is described as straight, cylindrical, and inelastic. Given these conditions, Poiseuille’s law can be applied to calculate the shear rate as follows:

(2) $$ỳ=\frac{8\cdot u}{d}$$

or

(3) $$ỳ=32\;\frac{Q}{\pi \cdot d^{3}}$$

In this context, ‘*u*’ is now the average velocity, ‘*Q*’ is the average volumetric flow rate, and ‘*d*’ is the channel diameter. These equations represent a parabolic velocity profile [23].

#### 2.1.2. Determination of Shear Stress

The magnitude of the shear stress on a planar surface caused by the flow of Newtonian fluids can be calculated using Newton’s law:

(4) $$\pi =µ\cdot \frac{du}{dy}$$

In the above equation, the symbol “µ” is used to represent the kinematic viscosity, while “*u*” is used to represent the fluid velocity. In addition, “*y*” is used to represent the distance from the surface and “*du*/*dy*” is used to represent the velocity gradient, also known as the shear rate ỳ ($s^{-1}$).

The Haagen–Poisseuille equation defines the shear stress for blood flow in a vessel (inelastic, cylindrical, and straight) as follows:

(5) $$\pi =32\cdot µ\cdot \frac{Q}{\pi \cdot d^{3}}$$

or

(6) $$\pi =8\cdot µ\cdot \frac{u}{d}$$

Here, *u* is the average fluid velocity. The shear stress increases in direct proportion to the number of the blood flow rate and decreases with the number of the vessel diameter [23].

#### 2.1.3. Circumferential and Axial Stress

Circumferential stress is a force that acts on blood vessels. It describes the perpendicular force exerted on the vessel wall by intraluminal pressure [24]. The radial tensile force produced by the pressure creates an internal circumferential (or hoop) stress in the vessel wall [25]. The dimensions of the vessel wall in both the loaded and unloaded conditions were used to determine the distribution of circumferential stress within the vessel wall [26]. The circumferential stress experienced by the vessel wall is proportional to its diameter. As the vessel diameter increases, the circumferential stress increases, provided the transmural pressure difference and wall thickness remain constant [27,28]. On the other hand, axial stress affects length adaptations in blood arteries and is determined by longitudinal force, radius, and wall thickness.

In mathematical–physical models of blood vessels, the “zero-stress state” of the vessel wall is typically established by referencing atmospheric pressure (approximately 750 mmHg = 100 kPa). This choice results in only positive circumferential and axial stresses, since the transmural pressure over the radial wall depth can only induce tensile stresses (assuming no residual stresses). However, an alternative definition of the zero-stress state using vacuum pressure (=0 mm Hg) includes the isotropic compressive stress (-pa) generated by atmospheric pressure throughout the wall. Consequently, negative (=compressive) stresses are possible. This distinction becomes critical when investigating the relationship between wall stress, vessel structure, growth, damage, and adaptation processes. A previous study examined axial, circumferential, and radial wall stresses using both conventional and unconventional zero-stress-state definitions for three sample vessels [29]. The results indicate that there is a tendency for axial wall stress to exhibit compressive characteristics in a significant number of vessels. In addition, there are significant differences between conventional and unconventional stress values, indicating that stress ratios are significantly influenced by the zero-stress-state definition used [29].

#### 2.1.4. Interaction between Hemodynamic Forces, Vascular Structure, and Vascular Response

The inherent inability to properly assess internal axial forces in living organisms limits the availability of in vivo data. Endothelial and vascular smooth muscle cell proliferation occurs in response to an increase in axial stress resulting from arterial stretch, whereas shear stress and circumferential strain remain unaffected. Increased MMP (matrix metalloproteinase activity, a family of zinc-dependent extracellular matrix proteins) and extracellular matrix (ECM) accumulation coincide with this process, resulting in compensatory length growth to restore normal axial tension [30,31,32]. In situations of laminar flow, shear stress is determined by factors such as blood viscosity, flow velocity, and vessel diameter. Shear stress increases as vessel diameter decreases, assuming viscosity and velocity remain constant. Typically, when subjected to increased shear stress, blood vessels tend to dilate to adapt and return to their normal conditions. Theoretically, vessels can expand and remodel indefinitely within the body’s limits as long as this results in normalization of circumferential strain and shear stress. However, the mechanical adaptability of elastin and collagen in the vessel wall is limited [24].

Wall stress is proportional to blood pressure, especially pulse pressure. Blood pressure exerts three types of stress on the arterial wall: longitudinal stress, radial or normal stress (perpendicular to the vessel axis), and tangential or hoop stress. Shear stress acts parallel to the surfaces of the intima, media, and adventitia layers, causing one layer to slide over the other. Strain (the amount of deformation per initial length of material) is related to stress. Shear stress can damage a vessel wall by damaging the intima (the inner layer of the vessel) as blood passes over the surface, and this is now thought to be the portal for plasma lipid uptake into the wall, leading to atherosclerosis [33]. However, the influence of shear stress does not stop at the intima. Shear stress can alter the inner layers of a multilayered artery, causing one layer to shift relative to another depending on the blood pressure and the different properties of each layer [34].

Changes in mechanical forces, such as stretch or shear stress, induce adjustments in the structure of the vessel wall to accommodate the changed conditions and ultimately return the tensile and shear stresses to their original levels [35,36]. Transient changes in vessel width occur as a result of acute changes in mechanical stress. These changes are primarily regulated by the release of vasoactive agonists or changes in myogenic tone. In contrast, chronic changes result in significant adaptive changes in the shape and composition of the vessel wall [37,38].

This process is referred to as vascular remodeling, which characterizes the changes that occur in vessels subjected to mechanical forces. For example, experimental hypertension leads to increased wall thickness in resistance arterioles and arteries due to vascular smooth muscle cell (VSMC) hyperplasia and in conduit arteries due to hypertrophy. Similarly, reduced mechanical stress leads to vascular atrophy [39].

### 2.2. Shear Stress Applied to Endothelial Cells In Situ

Shear stress is applied to endothelial cells by blood flow. It significantly affects vascular physiology and endothelial cell function. The effects of shear stress on arteries and its role in regulating endothelial cell behavior are discussed in this section [40].

The aorta and other large arteries are affected by laminar shear stress, which has the characteristics of unidirectional blood flow with limited spatial and temporal variability [41]. Arterial shear stress, which varies throughout the vasculature, is essential for maintaining endothelial function and integrity. Laminar shear stress, with unidirectional flow and moderate temporal and spatial variability, is a condition that affects large arteries such as the aorta. Endothelial permeability is affected, endothelial adhesion molecule expression is regulated, and the generation of vasodilators, including nitric oxide (NO), is stimulated, while thrombosis and endothelial inflammation are inhibited [15,42]. Expression of mechanosensitive genes associated with vascular remodeling and the development of atherosclerosis is influenced by pulsatile and bidirectional flow in medium-sized arteries [43]. Smaller arterioles have branched and tortuous shapes resulting in disturbed or oscillatory flow patterns. Different shear stress patterns in arteries have different effects on endothelial cell physiology and gene expression [15,41,42].

Capillaries are critical for nutrient exchange and tissue perfusion. They are subject to less shear stress than larger arteries. Shear stress in capillaries is typically minimal and exhibits significant spatial and temporal variability due to the variable distribution of blood flow [44]. Endothelial cell shape, alignment, and fenestration formation in capillaries change under shear stress. In addition, shear stress influences the development of transporters that aid in nutrient exchange and angiogenesis. The delicate interaction between shear stress and endothelial responses in capillaries is essential for tissue homeostasis and optimal microvascular function [40].

Activation of signaling pathways causes endothelial cells to respond differently to shear stress. Cell surface mechanosensors detect and transduce mechanical signals that initiate a series of events that control gene expression, cytoskeletal reorganization, and the production of vasoactive chemicals [45,46]. Vasodilation, inhibition of platelet aggregation, and reduced inflammation are all induced by activation of endothelial nitric oxide synthase (eNOS). In addition, shear stress activates ERK, p38, JNK, and MAPK signaling pathways that promote migration, proliferation, and inflammatory responses. Modifying transcription factors (KLF2, NF-B) control the expression of shear-stress-sensitive genes [47,48].

Vascular tone refers to the degree of constriction or relaxation in the walls of blood vessels, particularly arteries and arterioles. The relationship with NOS (nitric oxide synthase) lies in its role as a key regulator of vascular tone. Nitric oxide (NO) generated by ECs plays an important role in mediating the acute dilation of arteries that occurs when blood flow increases in these vessels [49]. Shear stress has been shown to increase nitric oxide production by activating endothelial nitric oxide synthase and upregulating its gene expression [50]. Shear stress has also been shown to stimulate NO production in cultured ECs [51]. Tetrahydrobiopterin, a key cofactor of eNOS, and intracellular Ca^2+^ levels increase in response to shear stress, and protein kinase activation turns on eNOS [52,53]. NF-κB, a shear stress response element in the eNOS gene promoter, and 3′ polyadenylation stabilize eNOS mRNA to enhance transcription in response to shear stress [54,55]. ECs exposed to shear stress also produce more of the potential vasodilators prostacyclin, adrenomedulin, and C-type natriuretic peptide [56]. Endothelin production and expression of the enzyme that converts angiotensin to the potent vasoconstrictor angiotensin II both decrease in response to shear stress [57].

#### 2.2.1. Sensing of Shear Stress at Cell–Cell Contacts in Endothelial Cells In Situ

Fluid shear stress is continuously applied to endothelial cells. Cell structure, cytoskeletal organization, biosynthetic activity, protein expression, and gene expression are all altered by this exposure. These adjustments are accompanied by changes in signaling activity, according to several in vitro studies. When a ligand binds to its receptor, signaling is ligand-dependent, but mechanical events can also trigger signaling by activating sensor molecules. The mechanism by which endothelial cells sense shear stress remains to be elucidated.

Tyrosine phosphorylation is known to be used in cell signaling to activate the catalytic activity of certain enzymes. Unfortunately, the biochemical techniques used in studies often do not provide spatial information due to their reliance on cultured cells. Imaging techniques, on the other hand, allow the localization of signaling events within specific cellular regions. In vivo research is difficult but necessary to validate data obtained from genetically engineered systems. Thus, a study was proposed in our laboratory to identify the sites of shear-stress-dependent signaling in normal endothelial cells. The authors coarctated blood vessels and analyzed endothelial cell shape changes as a function of aortic shear stress levels [58]. The study shows that cells exposed to higher shear stress exhibit elongated shapes, whereas those exposed to lower shear stress appear polygonal. Immunolabeling studies show that regions of high shear stress have higher levels of phosphorylated tyrosine (pY) proteins, whereas cells near the border of reduced ss regions have polarized staining [12]. This shows that pY-protein expression is locally regulated within cells. Researchers discovered two types of responses in endothelial cells: global changes in cell and nuclear structure and localized control of pY-protein expression. These findings suggest the existence of a shear stress sensing or signaling mechanism at cell–cell contacts within the intact endothelium, which may involve molecules such as PECAM-1 (platelet endothelial cell adhesion molecule-1) [7,59,60,61,62].

**Figure 1:**
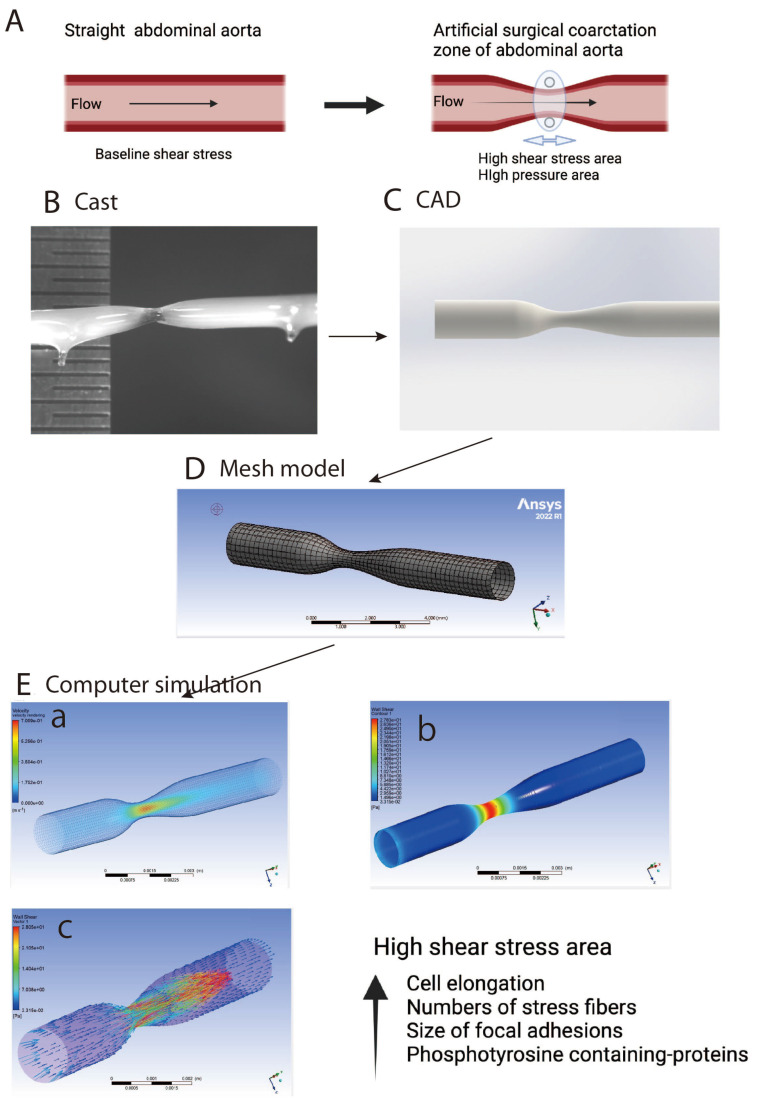
An area of induced shear stress at the site of surgical coarctation of the guinea pig abdominal aorta in situ. Surgical procedures for coarctation were described in our previous report [12]. To summarize, the abdominal aorta was exposed and gently constricted with a 0.5 mm diameter suture (**A**). The narrowest point of a constriction, determined at the time of en face specimen preparation, was 50–70% of the unconstricted area of the vessel. The peritoneum and abdominal wall were sutured separately and the animals were fed normally for 7 days when they were sacrificed and the abdominal aorta was dissected as described above. Shear stress simulation was performed as follows. First, a cast of coarctated abdominal aorta was made (**B**) and CAD model of abdominal aorta cast was created using SolidWorks (**C**) and rendered accordingly. Second, the CAD model was imported into Ansys Workbench (Education version of 2021R2) and a thorough geometry check was performed using the SpaceClaim module to identify possible errors in the geometry. The model was then edited and meshed to prepare it for simulation (**D**). The mesh file was then imported into the Ansys Fluent module. The inlet boundary condition was set with a velocity of 6.6 cm/s. At the outlet, a pressure difference of 40 mmHg was applied to allow the flow to pass. The simulation was iterated 100 times until the solution converged. In the post-processing phase, results were calculated that included velocity (**E-a**) and wall shear stress (**E-b**) and wall shear stress with vectors (**E-c**). Wall shear stress at coarctation area was 81.3 dyn/cm^2^ and the baseline of wall shear stress was 19 dyn/cm^2^. Figures were made with Ansys Workbench (Ansys Inc., San Jose, CA, USA). See also Kano et al. (2000) [12] for immunofluorescent microscopy. Supplemental videos are shown in Video S1.

The presence of mechanosensors in these regions is suggested by the presence of a different staining pattern in endothelial cells located at the boundary between low and high shear stress areas, according to experiments [12] (Figure 1E). Cells lining the flow divider were elongated in the direction of blood flow, consistent with the general notion that these cells are exposed to high shear stress. Immediately downstream of the flow divider was a patch of polygonal cells [12,63,64], which we believe corresponds to the area of reduced shear stress seen in the computer simulation. The abrupt cell shape transition from elongated to polygonal suggests a steep shear stress gradient in this cell shape transition region [46]. The size and shape of the low shear stress areas at the proximal and lateral locations changed significantly depending on the blood flow velocity values used in the calculation. However, at the distal location, the pattern of shear stress distribution was stable over a wide range of flow velocities. The author found that the configuration of coarctation area and shear stress intensity altered the endothelial cell shape and protein expression. The author further observed a characteristic change in cell shape and protein expression from elongated to polygonal near the coarctation area. They also discovered a polarized localization of pY-proteins within individual cells, with greater staining intensity on the side subjected to greater shear stress in both endothelial cells in situ [12] (Figure 1E).

The research further elucidated the flow sensing and signaling mechanisms of endothelial cells, suggesting that they have a flow sensing mechanism linked to cell–cell interactions that are active in their native environment. Endothelial cells constantly monitor and respond to variations in blood flow. These discoveries have provided important insights into the complex systems that control vascular function and blood flow throughout the body.

We have previously reported that pY-proteins are associated with cell–cell adhesion in in situ endothelial cells and that shear stress appears to enhance this association [12]. We hypothesize that endothelial cells within a region of reduced shear stress would express lower levels of pY-proteins. En face preparations of the distal opening of the intercostal artery were stained with anti-pY-proteins, serial optical sections were recorded using a confocal microscope, and a projected image was acquired using confocal laser scanning microscopy (CLSM). Stronger staining was generally found in the apical part of the cell border [12,63].

The authors designed a simpler blood flow pattern in the atherosclerosis model to avoid sample preparation issues. They found that endothelial cells subjected to greater shear stress near the constriction were axially elongated and stained more intensely for pY-proteins. Double staining with anti-PY and rhodamine-phalloidin using CLSM revealed increased staining of anti-PY in the surgical coarctation zone [12]. In addition, the high-power view using CLSM also revealed that anti-PY staining accumulated in the upstream half of individual endothelial cells, especially at the edge of the upstream portion of the cell. In addition, two types of SFs are observed on the apical surface of the endothelial cells. Conversely, cells in the reduced shear stress zone had a distinct shape and lower staining intensity for pY-proteins. The sudden shift in cell shape and pY-protein synthesis coincided with the narrowest point of the constriction (double stained with rhodamine-labeled phalloidin for F-actin visualization and b-stained with anti-pY antibody for tyrosine-phosphorylated proteins) [12].

In conclusion, shear-stress-induced changes in protein expression and localization within endothelial cells were detected. These results suggest that endothelial cells may locally adapt to different levels of shear stress. The researchers identified two possible mechanisms for polarized protein distribution: mechanical action mediated by shear stress and local shear stress sensing. Further research is needed to identify the specific proteins involved in these responses and to gain a better understanding of the underlying mechanisms.

#### 2.2.2. Sensing Shear Stress at Other Sites in Endothelial Cells In Situ

Blood vessels are dynamic organs that play an important role in maintaining the homeostasis of the circulatory system. They are not just tubes for the passage of blood; they are organs that play a role in the circulation of blood. The endothelial cells (ECs) that line the inside of blood vessels are constantly subjected to shear stress as blood moves through them. The responses of the endothelium to shear stress are thought to have a significant impact on blood-flow-dependent processes such as atherosclerosis and angiogenesis [33,42,65,66]. The way ECs respond to shear stress suggests that they are capable of sensing and transmitting information within the cell.

#### 2.2.3. Pathogenic Processes in Abdominal Aorta Aneurysm and Cell Signaling

In abdominal aortic aneurysm (AAA), the abdominal aorta swells to a size greater than 30 mm, or more than 50% greater than the diameter of the adjacent artery. This serious and potentially fatal vascular disease disproportionately affects men over the age of 65 [67].

Several pathogenic processes, including vascular smooth muscle cell (VSMC) death, oxidative stress, inflammatory and immunological responses, and extracellular matrix remodeling [68], have been implicated in the pathogenesis of AAA. The extracellular matrix (ECM) of the aortic wall is critical for vascular stability. Elastin, collagens, glycoproteins, and proteoglycans are the primary extracellular matrix (ECM) components of the aortic wall [69]. The ECM plays a role in controlling many vascular cell behaviors [70] and is responsible for the mechanical flexibility and stability of the vessel wall. AAA develops and worsens because of ECM remodeling, which is mainly characterized by the degradation of elastin and collagen [71]. In a study conducted by Didangelos et al. [72], a proteomic analysis was performed on human AAA samples. The results showed changes in the expression levels of several proteins, including fibronectin, tenascin, thrombospondin 2, periostin, and collagen XII. Changes in the extracellular matrix (ECM) profile have been linked to both larger aortas and problems with normal body function [73]. Endothelial mechanotransduction is a process by which endothelial cells sense and respond to mechanical stresses. Endothelial cells and an inflammatory response in the aortic wall can be triggered by disturbed blood flow patterns typically associated with low and oscillating shear stress. Inflammatory cells and molecules can invade the vessel wall. Degradation of the extracellular matrix and weakening of the aortic wall are critical to the development of AAA. Endothelial dysfunction is characterized by impaired nitric oxide production and increased production of reactive oxygen species (ROS) after prolonged exposure to abnormal shear stress patterns [74,75]. The pathogenesis of AAA may be caused by this imbalance, which can lead to oxidative stress, inflammation, and vascular remodeling [75,76].

Signaling through mitogen-activated protein kinases (MAPKs) is critical for controlling a wide range of cellular responses in response to a broad spectrum of environmental stimuli. MAPKs include the p38 family of kinases (p38 MAPK), the ERK family of kinases (ERK1/2), and the Jun amino-terminal kinases (JNK1/2/3). DiMusto et al. [77] found elevated levels of phosphorylated and total JNK in human and rodent AAA tissues. There are two pathways by which JNK signaling controls ECM metabolism in AAA. One line of evidence suggests that JNK activation promotes ECM degradation during AAA pathogenesis by increasing MMP production, specifically MMP9 from THP-1 monocyte–macrophages and Mmp2 from rodent VSMCs [78]. Furthermore, AngII-induced AAA in mice is characterized by increased expression of Mmp2 and Mmp9, but not MMP1, due to the pro-inflammatory effects of cigarette smoke (or nicotine) [79]. When critical ECM-synthesizing enzymes are downregulated by JNK signaling, both ECM production and tissue healing are suppressed. JNK activation reduces the expression of lysyl oxidase (Lox), which forms aldehyde residues and crosslinks elastin and collagen, and poly-4-hydroxylase I (P4ha1), which synthesizes, secretes, and deposits collagen [80].

Several physiological and pathological processes are controlled by nuclear factor-kB signaling. In addition, several NF-κB /Rel proteins are involved in this signaling pathway. These include c-Rel, Rel B, Rel A, p52, p50, NF-κB2, and NF-κB1. These proteins act as transcription factors by forming dimers and binding to specific regions of DNA. NF-κB controls the expression of many MMP genes [81]. Upregulation of Mmp12, Mmp3, Mmp9, and Mmp2 by NF-κB signaling contributes to the progression of AAA in elastase-infused rats [82]. An NF-κB inhibitor, a chimeric decoy oligodeoxynucleotide (ODN), was administered to elastase-perfused animals. The expression of MMP12, MMP9, MMP3, and MMP2 was found to be significantly decreased by ODN therapy, and AAA production was also prevented [83].

The transforming growth factor β, associated with signaling pathways, plays a critical role in the regulation of various biological processes. Signaling is initiated by the binding of TGFβ ligands, namely TGFβ1, TGFβ2, and TGFβ3, to TGFβ receptors, which include TβRI, TβRII, and the coreceptor TβRIII. The two separate pathways activated by TGF signaling are the SMAD-dependent pathway, also known as canonical signaling, and the SMAD-independent pathway, also known as noncanonical signaling. The noncanonical pathway includes a variety of cascades, including the MAPK and RhoA cascades. Canonical signaling refers to a cellular process in which the TβRI/TβRII complex phosphorylates downstream SMAD2/SMAD3 proteins. This phosphorylation event triggers the recruitment of SMAD4 and the subsequent translocation of the protein complex to the nucleus. The primary goal of this translocation is to exert control over transcriptional processes [84].

Previous studies have documented the occurrence of decreased expression levels of TβRII and SMAD3, critical components of the transforming growth factor β (TGFβ) signaling pathway, in tissues affected by abdominal aortic aneurysm (AAA) in humans [85]. In their study, Dai et al. [86] investigated the effects of TGFβ signaling on a rat model of abdominal aortic aneurysm (AAA) created by preformed xenografts. They used adenoviral delivery of active TGFβ1 to investigate its involvement in this context [86]. Excessive activation of TGFβ signaling prevented aortic enlargement and preserved the integrity of the elastin architecture. At the same time, overexpression of TGFβ1 resulted in a reduction of Mmp9 and Mmp2 levels while facilitating tissue healing characterized by a substantial presence of collagen and elastin.

In another study, the effect of using a TGFβ neutralizing antibody to block the pathway was investigated. The results showed that this intervention led to stimulation of aneurysm rupture via MMP12 in mice with AAA induced by AngII infusion. In addition to TGFβ1, the involvement of downstream SMAD proteins and TGFβ receptors in the preventive mechanisms against AAA has been observed [87]. The conditional gene has been used to knockout mice in conjunction with AngII infusion to find that deficits in TβRII and Smad4 led to the development of severe AAA and increased levels of Mmp12 and cathepsin S, while the levels of Mmp9 or Mmp2 remained unaffected [88]. In another study, the development of CaCl2- and AngII-induced AAAs was significantly enhanced in mice lacking the Smad3 gene. This was accompanied by increased expression of Mmp2, Mmp9, and Mmp12, in addition to collagen fiber disruption and elastin fragmentation in the aortas [89].

#### 2.2.4. Turbulent Flow at the Bifurcation of the Carotid and Iliac Arteries

Womersley [90] demonstrated that blood flow in the primary arteries can be accurately described by a one-dimensional, time-dependent solution of the Navier–Stokes equations. This solution allows the characterization of blood flow using a Fourier decomposition of the cardiac harmonics [90]. Wall elasticity and non-Newtonian blood viscosity were later incorporated into this model [91,92]. Modern studies of blood hemodynamics have relied heavily on the Womersley flow model (WFM). The WFM has led researchers to believe that blood flow is predominantly laminar and that a change in turbulence alters blood hemodynamics and may trigger the development of vascular diseases such as cerebral atherosclerosis and aneurysms. In order to characterize the hemodynamic patterns that govern the mechanobiology of endothelial cells, it is critical to correctly identify blood flow regimes [93]. Particle imaging velocimetry (PIV) has recently been used to demonstrate the presence of turbulence in pulsatile multiharmonic flow with a mean Reynolds number of 300, which is an idealized model of intracranial aneurysm flow [94]. Turbulence has been shown to add complexity to the hemodynamics of both intracranial aneurysms and carotid occlusive disease [95]. Carotid stenosis and atherosclerosis are known to be associated with disturbed blood flow [96,97].

Fluid shear stress alters vascular cell phenotype and gene expression. For example, heart tube development in zebrafish embryos is affected by fluid shear stress at the epigenetic level [98]. The aberrant development of ventricles and valves has been attributed to a lack of blood flow. The embryonic heart responds to both steady and perturbed flow. The direction of fluid shear stress within the node establishes left–right asymmetry in mouse embryos. In response to fluid shear stress, human bone-marrow-derived progenitor cells, mesenchymal progenitor cells, and embryonic stem cells can be induced to develop into endothelial cells.

The study of fluid shear stress is of great importance across multiple disciplines, encompassing areas such as developmental biology and cardiovascular health [99]. The effects of shear stress on vascular cells are dynamic in both space and time [100]. Endothelial cell activity is enhanced by the increased fluid shear stress induced by exercise [101]. The generation of pulsatile flow, including oscillatory shear stress, on straight arterial segments or the medial wall of bifurcations is atheroprotective, whereas the generation of turbulent flow on the lateral wall of arterial bifurcations is atherogenic [102]. Atherosclerosis, the location of abdominal aortic aneurysms, vessel wall remodeling, and high-risk coronary atherosclerotic plaques can all be predicted by identifying arterial zones of low wall shear stress (WSS) [103,104].

**Figure 2:**
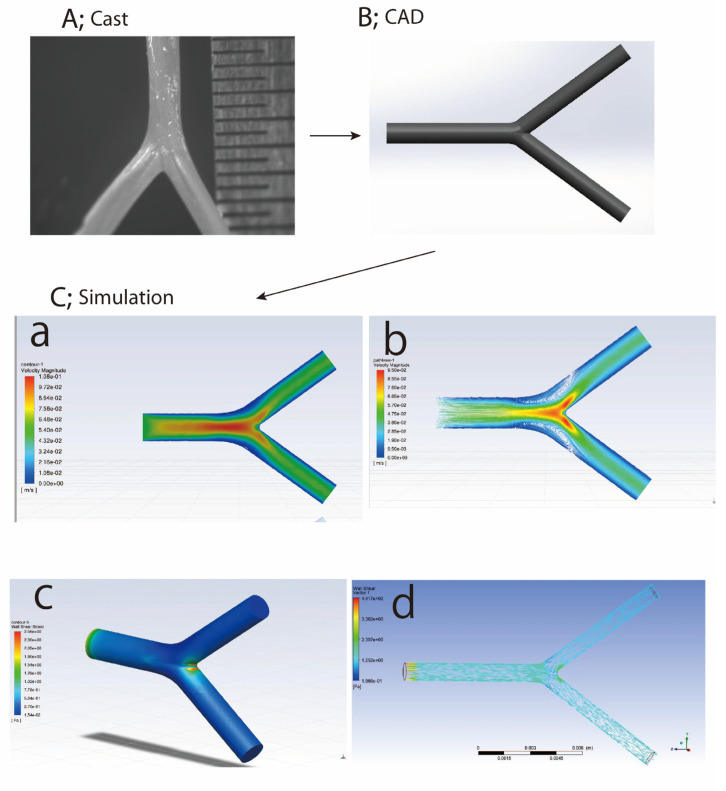
An area of induced shear stress at the bifurcation point of the guinea pig iliac artery downstream of the abdominal aorta shown in Figure 1. The simulation was performed using the same procedure as in Figure 1. A cast of the iliac artery was made (**A**) and a CAD model of the iliac artery was created and rendered (**B**). The CAD model was imported into Ansys Workbench and a thorough geometry check was performed using the SpaceClaim module to identify possible errors in the geometry. The results were displayed as velocity contour (**C-a**), velocity stress line (**C-b**), and wall shear stress of the vessel surface (**C-c**) and wall shear stress of vector (**C-d**). Figures were made with Ansys Workbench (Ansys Inc., San Jose, CA, USA). Supplemental videos are shown in Videos S2–S4.

The iliac artery bifurcation is where the common iliac artery divides into two Y-shaped branches (Figure 2). This is a common site for blood flow turbulence to develop due to the complex hemodynamic interactions at this junction. It is generally accepted that hemodynamic variables are critical in the development of arterial disease. In particular, it has been amply demonstrated that WSS, a critical regulator of endothelial function, is associated with the onset and development of atherosclerosis [105]. Previous studies have shown that elevated levels of WSS often occur at vessel bifurcations or regions of articulated flow or bending [106]. This underscores the critical influence of vessel morphology on nearby hemodynamic conditions.

In addition, blood flow pulsations show periodicity at rest [107] (Figure 2). A zone of distribution occurs only distal to the renal branches along the downstream walls of the aorta during periods of rest, resulting in low WSS values [108]. On the other hand, regions described with low shear stress and high oscillatory shear index generally decrease with moderate exercise [109]. In a comparative study by Khader et al. [110], they analyzed healthy and idealized stenotic renal arteries under exercise and resting conditions. The authors found zones of distribution on the proximal side at rest, which diminished after exercise. They also found that WSS patterns at the renal bifurcation were more articulated on the distal side and within the stenotic area during exercise compared to resting conditions [110].

### 2.3. Cyclic Strain

Cyclic strain, particularly in arteries and heart valves, is the cyclic deformation of blood vessels caused by pulsatile blood flow. The vessel wall is periodically stretched and relaxed by this mechanical stress, causing the endothelial cells to respond [15].

Blood flow induced by myocardial spasm and relaxation creates cyclic tension on arterial walls. The amplitude and duration of cyclic stretch are influenced by blood pressure, vessel diameter, and compliance [111]. Other studies have shown that cyclic strain affects endothelial cell proliferation, migration, and release of vasoactive substances. It also affects vascular smooth muscle cell junctions, inflammation, extracellular matrix remodeling, and gene expression. Stretch-activated ion channels, focal adhesion complexes, and integrins are examples of mechanosensitive proteins that mediate the response of arterial endothelial cells to cyclic stretch [112,113].

Blood flow patterns throughout the cardiac cycle place cyclic stress on the heart valves. The opening and closing of the valves exert mechanical stresses on the endothelial cells, including tensile strain and bending deformation. The integrity and functionality of the heart valves depend on these stresses [114]. Cyclic loading of the heart valve affects the phenotype, alignment, and remodeling of endothelial cells and the extracellular matrix. It regulates the production of proteins such as matrix metalloproteinases (MMPs), transforming growth factor-beta (TGF-), and bone morphogenetic proteins (BMPs), which are involved in valve remodeling and calcification. In addition, according to Balachandran et al. [114], interstitial valve cell activation and inflammation, both of which play a key role in the pathogenesis of valvular diseases, including calcific aortic valve disease and valve dysfunction, are under the control of cyclic loading.

Cellular responses to cyclic strain involve mechanisms called mechanotransduction pathways. Two of these pathways, integrin and focal adhesion complex activation, transmit mechanical signals through the extracellular matrix to the cytoskeleton and trigger metabolic processes. The MAPK cascade (p38, ERK, JNK) is activated by cyclic stress, which controls cell proliferation, produces inflammatory genes, and is involved in cell death [1]. Cyclic stress leads to the production of endothelin-1 (ET-1), prostaglandins, NO, and vasoactive substances that influence inflammation and vascular tone. The transcription factors NF-B and AP-1 modify gene expression in response to cyclic stress signals. In addition, DNA methylation and histone acetylation are affected by cyclic stress and contribute to long-term biological responses when cells are physically challenged [48].

### 2.4. Hydrostatic Pressure

It is well known that cells sense and respond to a variety of mechanical stimuli, including hydrostatic blood pressure and shear stress [115]. The advantageous location of vascular ECs allows them to detect changes in blood pressure and other blood-borne signals. In response, they can release vasoactive substances. Under physiological conditions, vascular homeostasis is easily maintained in favor of vasodilation due to the balance between endothelium-derived relaxing and contracting factors [116]. ECs control blood flow to tissues jointly with vascular smooth muscle cells, as well as various components that circulate in and out of tissues and local vasoregulation [117]. In addition, the endothelium is involved in the conversion and degradation of vasoactive agents and the ECs are responsive to these agents. They modify their metabolism according to their needs and the oxygen tension of the tissues. Vasodilators such as prostacyclin (PGI2) and nitric oxide (NO) are produced and released by the ECs, which is important for keeping the heart in balance [118].

Hydrostatic pressure is the pressure exerted by a column of fluid, such as blood, within blood vessels. The circulatory system relies on hydrostatic pressure to control blood flow and provide optimal perfusion to tissues and organs. Hydrostatic pressure in blood vessels varies depending on factors such as the location of the vascular tree and the degree of pathological disturbance. Gravity causes hydrostatic pressure to increase in the lower extremities. In pathological conditions such as hypertension, the hydrostatic pressure may increase and lead to endothelial dysfunction and vascular damage [119].

Endothelial dysfunction, characterized by impaired vasodilation, increased oxidative stress, inflammation, and increased vascular permeability, can be caused by elevated hydrostatic pressure, which may also affect other cell types. Hydrostatic-pressure-induced endothelial dysfunction is associated with several disease-related changes. ET-1 and NO synthesis and release are affected, leading to an inappropriate regulation of vascular tone [120]. In addition, oxidative stress and ROS (reactive oxygen species) are increased after an increase in hydrostatic pressure, both of which cause inflammation and damage to the endothelium. In addition, the production of adhesion molecules is altered and leukocyte adhesion to the endothelium is enhanced, leading to an inflammatory response [121].

Endothelial cells are affected by hydrostatic pressure through several signaling mechanisms [122,123,124]. Hydrostatic pressure causes calcium influx and opens ion channels, including transient receptor potential (TRP) channels and mechanosensitive receptors. An important mechanism triggered by hydrostatic pressure is the RhoA/ROCK (Rho-associated protein kinase) system, which controls endothelial cell adhesion, cell contractility, and cytoskeletal rearrangements [125,126]. In contrast, pro-inflammatory genes and adhesion molecules are upregulated when the NF-B pathway is activated [127,128].

Therapeutic targeting is enabled by the identification of molecular processes and signaling pathways involved in cellular responses to mechanical stress [48]. Regulation of these pathways may improve cardiovascular health, restore endothelial function, and prevent or reverse vascular remodeling.

Pharmacological treatments involving integrins, downstream signaling molecules, and mechanosensors have shown promise in preclinical studies. For example, NO bioavailability and endothelial function can be improved by drugs that increase eNOS activity or reduce oxidative stress [129]. The use of mechanical therapies, such as mechanical stimulation or tissue engineering techniques, in addition to pharmacological treatments is a novel approach that can mimic natural mechanical stresses and promote endothelial repair and regeneration.

## 3. Mechanical Stress Applied to Cultured Endothelial Cells In Vitro

### 3.1. Endothelial Cell Culture Techniques

Consideration of the potential impact of mechanical forces on cell morphology and physiology is a critical factor when initiating cell-based assays with endothelial cells. Many investigators have attempted to cultivate individual intimal cells or entire segments of the arterial wall to obtain endothelial cells in vitro. However, these technologies have not been widely adopted due to ambiguity regarding the identity of the cultured cells or inadequate maintenance of cell growth and viability. Jaffe and colleagues recently published a study on the primary culture of human umbilical vein endothelial cells that could be identified by several morphological and immunological parameters. A long-term culture of vascular endothelial cells is now possible due to recent developments in cell culture methods [130]. Endothelial cells from large blood vessels, such as bovine or porcine aorta and human umbilical vein, have been the source of most success to date. The endothelium of a bovine aorta has just been cloned. Bovine aortas have also been used to obtain arterial endothelial cells by mild collagenase treatment and medium perfusion [131].

Cell culture methods are essential to study the effects of mechanical stress on endothelial cells in a reproducible and controlled manner [132]. An overview of the various in vitro cell culture methods is provided in this subsection. The most basic and commonly used cell culture models are monolayer cultures [132]. Endothelial cells are grown in a monolayer on tissue culture plates or dishes, making them easier to manipulate and observe. In a more physiologically relevant environment, endothelial cells are co-cultured with other cell types, such as vascular smooth muscle cells or immune cells, which can be used to study cell-to-cell interactions [133,134,135,136,137,138].

Cells are cultured under flow for hours to several weeks to achieve a more physiological and in vivo state, resulting in more relevant results. Simply put, flow conditioning is essential for any study involving cells that are exposed to physiological flow conditions. In vitro fluidic systems, particularly those used to apply shear stress to endothelial cells, are critical tools in biomedical and biological research. These systems are designed to mimic the mechanical forces that blood flow exerts on the inner lining of endothelial cells. There are several recent in vitro fluidic systems used to apply shear stress to endothelial cells: parallel-plate flow chambers, cone-and-plate devices, orbital shakers, and microfluidic systems. In parallel-plate flow chambers (PPFCs), two plates are placed in parallel to provide an open channel at either end for fluid flow [139]. Recently, dynamic studies of cell adhesion with well-defined shear forces have been performed using commercially available parallel-plate flow chambers [140,141,142,143]. The ability to operate with limited quantities of cells and/or reagents is possible using various seal or plastic-plate thicknesses of the experimental design [139,144]. The cone-and-plate system was originally invented as a rheometer and has been used for rheological measurements on non-Newtonian fluids for many years [145,146]. The orbital shaker or “swirling well” method involves culturing cells in a culture plate on a shaker platform, where the movement of the platform causes circular motion of the fluid in the well [147,148,149]. This fluid motion applies multiaxial and uniaxial shear stress to the cells cultured at the center and edge of the well bottom, respectively [149]. Microfluidic devices that can simulate shear stress under real mechanical conditions in vivo have been widely used [150,151].

In recent years, three-dimensional (3D) models have become increasingly popular. These models represent the natural tissue architecture and microenvironment more accurately. This can be achieved using methods such as scaffold-based cultures or spheroid/organoid cultures. The study of complex biological responses to mechanical loading is enabled by the addition of multiple cell types and extracellular matrix elements to 3D specimens [126,152].

### 3.2. Application of Shear Stress

Shear stress is one of the most widely studied mechanical stresses on endothelial cells in vitro. It can be applied to cultured cells in several ways to study biological responses and signaling cascades induced by shear stress. Static and dynamic shear are the two approaches used to study shear stress. Under static shear, cells are exposed to a constant flow, which is advantageous for rapid biological responses such as changes in gene expression or protein synthesis. To study mechanotransduction pathways, long-term cellular responses, and shear stress adaptation, dynamic shear mimics pulsatile blood flow [132,153].

Studies of endothelial cell responses to mechanical stress have been transformed with microfluidic technology. These devices use microscale channels to create regulated flow patterns and precisely expose cells to shear stress. They enable the study of different cell types, using 3D culture models, and precisely adjusting shear stress parameters. Microfluidics enable the study of real-time monitoring of cell behavior, active biological responses, and high-throughput drug screening under shear stress conditions [153,154]. Microfluidic tools have revolutionized the study of shear stress, providing insight into the mechanobiology of endothelial cells and their response to flow-induced mechanical stressors. By using these in vitro techniques, researchers can better understand the cellular and molecular responses of endothelial cells to mechanical stress, elucidate primary mechanisms, and identify novel therapeutic targets for cardiovascular disease [132,153,154].

### 3.3. Mechanical Strain Models

Uniaxial strain models mimic physiological or tissue-specific conditions by applying mechanical stresses that stretch cells along a single axis. The current strain is obtained using specialized equipment such as mechanical stretchers or stretch chambers. Uniaxial stretch experiments demonstrate effects on endothelial cell morphology, cytoskeletal structure, alignment, gene expression, and behavior. Changes in adhesion molecules, inflammatory mediators, and extracellular matrix elements also affect migration, proliferation, and angiogenesis processes. Uniaxial strain studies are advancing our knowledge of vascular remodeling, tissue development, and wound healing [155].

Biaxial strain models use mechanical forces to stretch cells along two perpendicular axes, allowing researchers to study complex biological responses under physiologically realistic conditions. Strain is created using specialized technologies such as modified culture dishes or flexible membranes. Biaxial stretch affects endothelial cell activities such as alignment, migration, and proliferation. It also affects focal adhesion formation, cytoskeletal architecture, and expression of mechanosensitive genes. The maintenance of tissue homeostasis and vascular development also affects angiogenesis and the endothelial barrier function [156].

Stretchable substrates provide a flexible platform for studying how cells respond to mechanical stress. The materials, including elastomers and hydrogels, can deform in response to mechanical forces. These substrates provide controlled strain magnitudes and patterns, allowing the study of different biological responses. They have all been used to study endothelial cell adhesion, migration, angiogenesis, and mechanotransduction processes. According to Zhang et al. [113], stretchable substrates work well with various cell culture techniques and provide insightful data on the effects of mechanical strain on cellular activity.

### 3.4. Hydrostatic Pressure in Cell Culture

Pressure-driven devices are used to apply hydrostatic stress to cells cultured in vitro. These systems feature specialized bioreactors or chambers that generate and regulate hydrostatic pressure within the culture environment. Cells are often subjected to elevated pressures by adjusting the flow rate or height of fluid columns. Pressure-controlled devices allow researchers to study how hydrostatic pressure affects biological responses, such as changes in gene expression, cell structure, and protein release. They provide a controlled environment to study how different levels and durations of pressure affect endothelial cell behavior. According to Reinwald et al. [157] and Zvicer and Obradovic [158], these devices are useful for studying the consequences of elevated hydrostatic pressure, which is present in conditions such as tissue compression and hypertension.

Hydrostatic pressure affects the behavior and efficiency of endothelial cells, in particular, their shape, orientation, and cytoskeletal architecture. In addition, according to Ohashi et al. [159] and Charbonier et al. [160], hydrostatic pressure affects the expression of genes that control vascular tone, extracellular matrix remodeling, and inflammation.

## 4. Cellular Response to Mechanical Stress

### 4.1. Endothelial Barrier Function

Vascular integrity is maintained and the exchange of substances between the circulation and surrounding tissues is controlled by the endothelial barrier. The endothelial barrier function can be affected by mechanical stresses such as tension, shear stress, and hydrostatic pressure [161].

The integrity of the endothelial barrier is largely maintained by two distinct types of junctional complexes: adherens junctions and tight junctions. Adherens junctions provide cell–cell adhesion and mechanical stability, whereas tight junctions act as a physical barrier that limits paracellular permeability [162]. By controlling the formation and disassembly of junctional complexes, mechanical stress can affect the endothelial barrier function. For example, shear stress promotes tight junction maturation and growth, resulting in greater barrier integrity. Conversely, cyclic stress can damage the proteins that make up tight junctions, increasing permeability [163].

Mechanical stress affects endothelial permeability; in particular, shear stress decreases permeability by promoting tight junction formation and reducing adhesion molecule expression [164,165]. Conversely, extreme shear stress or long-term exposure to cyclic strain can increase permeability and disrupt the endothelial barrier. Changes in junctional proteins, cytoskeletal structure, and endothelial glycocalyx lead to these abnormalities [164,165,166]. Investigation of the processes that regulate endothelial barrier function under mechanical stress is necessary to understand vascular diseases associated with increased permeability.

### 4.2. Endothelial Nitric Oxide Synthase (eNOS) and Nitric Oxide (NO) Production

The activity of eNOS and NO production in endothelial cells is controlled by mechanical stress, particularly shear stress. NO, a potent vasodilator and signaling molecule with multiple physiological functions, is produced when shear stress activates eNOS [167,168]. Due to the phosphorylation of eNOS at specific sites induced by shear stress, NO is produced as a result of its activation. NO diffuses into the smooth muscle cells of the vasculature, causing relaxation and vasodilation. According to Sriram et al. [168] and Boo et al. [169], this contributes to the control of vascular tone, blood flow, and the maintenance of endothelial function. In addition, NO has several beneficial effects on the endothelial function, including anti-inflammatory and antithrombotic properties. It decreases the production of adhesion molecules, prevents platelet aggregation, and prevents leukocyte adhesion to endothelial cells [170,171,172,173]. NO also affects endothelial cell migration, angiogenesis, and proliferation [174]. NO is a signaling molecule that has multiple functions in physiological processes such as vasodilation (relaxation of blood vessels), neurotransmission, and immune responses [175,176,177,178]. Endothelial NOS (eNOS) and neuronal NOS (nNOS) are types of NOS that are mechanosensitive, meaning that they can respond to mechanical forces. For example, in the context of blood vessels, shear stress caused by blood flow can activate endothelial NOS (eNOS), leading to the production of nitric oxide, which in turn contributes to vasodilation. This is a mechanism by which blood vessels regulate blood flow based on mechanical cues. Tyrosine phosphorylation is a post-translational modification in which a group of phosphates is added to a tyrosine residue in a protein. The effects of this modification can be profound on protein function and cellular signaling. NOS and tyrosine phosphorylation are not directly related in the context of NOS function as a mechanoresponder. Some cellular processes may have indirect connections.

Nitric oxide (NO) is an important component of the vasculature, relaxing vascular smooth muscle and controlling blood pressure and vascular resistance [179]. Endothelial cells (ECs) produce NO in response to mechanical signals such as shear stress and pressure inside the cell [176]. When ECs are mechanically stimulated, they initiate a complex chain of metabolic events involving many mechanosensors and enzymes in the cells. The main goal of this chain reaction is to promote the enzyme endothelial nitric oxide synthase (eNOS), which helps to convert arginine (L-Arg), an α-amino acid, to NO [180]. Endothelial NO synthase (eNOS) is triggered by various agonists and fluid shear stress through a variety of cellular mechanisms, including increased intracellular Ca^2+^, interaction with substrates and cofactors, protein phosphorylation, interaction with adaptor and regulatory proteins, and shuttling between different subcellular domains [181]. Specific serine and threonine residues are phosphorylated by PKA and Akt. Activation of eNOS is dependent on its phosphorylation. eNOS is typically present as a monomer in an inactive state. The dimerization of eNOS is caused by the binding of phosphorylation and calcium-calmodulin, resulting in the formation of an active enzyme [182].

Mechanical forces can trigger the activation of tyrosine kinases, enzymes that add phosphate groups to tyrosine residues. The activity of NOS or other related molecules can be affected by downstream signaling pathways that may be triggered. In addition, NO itself can affect cellular processes involving tyrosine phosphorylation, as NO can react with certain molecules to form nitrosylated derivatives that can affect protein function [183,184,185].

It is worth noting that the relationship between NOS, mechanoresponsiveness, and tyrosine phosphorylation may be influenced by context and may involve complex signaling pathways that are still under investigation.

### 4.3. Inflammatory Responses

Shear stress in the arterial circulation has been estimated to range from negative values, through zero values at the edges of flow separation regions, to values of 40–50 dyn/cm^2^ based on many fluid dynamic studies and measurements and observations in real arteries. The shear stresses of a typical vessel are as follows: aorta—1–22 dyn/cm^2^ [186], arteries—10–70 dyn/cm^2^ [187], veins—1–6 dyn/cm^2^ [188], and capillaries—3–95 dyn/cm^2^ [189]. Transients in excess of 100 dyn/cm^2^ have been reported, suggesting that these values may increase significantly during periods of increased cardiac output or hypertension. Because it is easier to manipulate the mechanical environment in vitro than in vivo, several studies of mechanotransduction of hemodynamic forces have been performed there. Although the effects of pulsatile flow have recently received increasing attention, most in vitro studies have focused on shear stresses between 0 and 100 dyn/cm^2^ in constant unidirectional laminar flow [190,191,192,193,194].

By activating inflammatory signaling pathways, endothelial cells can produce and release adhesion molecules, chemokines, and pro-inflammatory cytokines in response to mechanical stress. The endothelial cell defense system against pathogenic and mechanical stress includes the inflammatory process [195]. Pro-inflammatory transcription factors such as nuclear factor-kappa B (NF-κB) are stimulated by shear stress and pro-inflammatory genes are upregulated. NF-κB is associated with nuclear localization and plays an important role in cellular responses to various stimuli, including mechanical forces. NF-κB is a transcription factor that regulates the expression of a wide range of genes involved in immune response, inflammation, cell survival, and other processes. Its involvement in mechanotransduction highlights its role as a nuclear determinant of cellular responses to mechanical stimuli [128,196,197]. These genes produce cytokines, including tumor necrosis factor-alpha and interleukin-6, chemokines, and adhesion molecules, including vascular cell adhesion molecule-1 and intercellular adhesion molecule-1, which help attract leukocytes and initiate an inflammatory cascade [195,198,199].

In addition, mechanical stress can trigger reactive oxygen species (ROS) produced by endothelial cells, leading to oxidative stress and activation of redox-sensitive pathways that promote inflammation. The initiation and development of vascular problems are influenced by the interaction of mechanical stress, inflammatory processes, and oxidative stress [200,201].

A pro-inflammatory state within the endothelium can be caused by low or disturbed shear stress, which occurs in regions of blood vessels with turbulent or oscillatory blood flow. Inflammation can be promoted by different levels of shear stress depending on various factors such as vessel type, individual differences, and specific conditions. Shear stress levels below 4 dyn/cm^2^ are generally considered low and may be associated with a pro-inflammatory response. Shear stress levels above 10 dyn/cm^2^ are generally considered to be more protective and anti-inflammatory [202,203].

### 4.4. Cell Proliferation and Apoptosis

Endothelial cell growth and death are influenced by mechanical stress; vascular remodeling is supported and contributes to the maintenance of tissue homeostasis. Cyclic strain, shear stress, and hydrostatic pressure have the ability to control cell cycle progression, cell survival, and cell proliferation [2,3]. As a result of cell cycle arrest induced by shear stress, endothelial cell growth has been shown to be inhibited. It promotes the growth of cell cycle regulators, including p21 and p27, while inhibiting and arresting cell cycle progression. The antiproliferative effect of shear stress helps maintain endothelial quiescence and vascular integrity [3].

Conversely, hydrostatic pressure and cyclic strain can promote endothelial cell development. Cyclic strain promotes cell proliferation by activating signaling pathways involved in DNA synthesis and cell development. Hydrostatic pressure can promote endothelial cell proliferation, which contributes to neovascularization and vascular remodeling, especially in pathological conditions [2,5]. Microvascular rarefaction refers to a reduction in the number and density of microvessels in various tissues and organs [204,205]. It results in the loss of arterioles and capillaries, which is the primary cause of hypertension, rather than promoting neovascularization (the formation of new blood vessels) [206]. This rarefaction typically involves a reduction in the number and density of existing small blood vessels within tissues and organs. Endothelial cell apoptosis, which is critical for maintaining protective tissue homeostasis and removing damaged cells, can also be affected by mechanical stress. In endothelial cells, shear stress induces resistance through apoptosis, which promotes cell survival. On the other hand, changes in mechanical pressure or pathological conditions can lead to endothelial cell death, resulting in endothelial dysfunction and vascular damage [3,207].

### 4.5. Extracellular Matrix Remodeling

Mechanical stress strongly controls endothelial cell extracellular matrix (ECM) remodeling. Kruger-Genge et al. [14] reported that elastin, collagen, and proteoglycans, among other ECM components, are produced, rearranged, and degraded during ECM remodeling. Endothelial cells respond to shear stress and cyclic strain by increasing their activity and synthesis of matrix metalloproteolytic enzymes (MMPs) and tissue inhibitors of metalloproteinases (TIMPs). The activity of MMPs is inhibited by TIMPs, which cause degradation of the ECM; the balance between degradation and creation of the ECM is kept constant [14,165].

Angiogenesis, wound healing, and tissue remodeling depend on mechanically driven ECM remodeling. Arterial sprouting and neovascularization processes influence endothelial cell behavior, tube formation, and migration. Endothelial cell communication with pericytes and vascular smooth muscle cells is also affected by ECM remodeling [14,165].

## 5. Molecular Mechanisms and Signaling Pathways

### 5.1. Shear Stress Mechanotransduction

Shear stress mechanotransduction is a topic that has been the focus of extensive research, although much about it is still unknown [190]. Based on the results of various studies, several pathways appear to be involved in the transduction of the shear stress signal.

Since the first shear stress sensor has not been found, it is unknown as to which signaling pathways are primary and which are secondary. Shear stress sensing has been shown to involve several types of membrane molecules and microdomains of cells, including G-proteins, ion channels, adhesion proteins, primary cilia, caveolae, the cytoskeleton, growth factor receptors, and the glycocalyx [208].

#### 5.1.1. Ion Channels

Many types of ion channels have been proposed as potential shear stress sensors. Shear stress causes potassium ion channels to open, resulting in hyperpolarization of the plasma membrane [209,210,211]. In contrast, shear stress causes chloride ion channels to open, depolarizing the membrane [212]. Certain Ca^2+^-permeable cation channels have been observed to respond to shear stress, resulting in their activation and subsequent facilitation of extracellular Ca^2+^ entry across the plasma membrane. Such channels include P2X purinoceptors and transient receptor potential (TRP) channels, both of which are produced by endothelial cells [213,214]. Ca^2+^-dependent signaling pathways are then activated by Ca^2+^ influx, causing the EC to respond to shear stress [190], and have been linked to endothelial Piezo1 channels [215,216]. Shear stress is transmitted through endothelial Piezo1 channels, a critical mechanism in vascular physiology [217]. Mechanical forces, such as the shear stress exerted by blood flow on the endothelial cells lining blood vessels, can cause activation of these mechanically activated ion channels. Shear stress causes Piezo1 channels to open, resulting in the influx of calcium ions (Ca^2+^) into the endothelial cells [218].

Shear stress on cultured ECs results in a dose-dependent increase in intracellular Ca^2+^ concentration [219]. The P2X4 cation channel subtype of the ATP-gated P2X purinoceptor is involved in the extracellular Ca^2+^ influx that causes the Ca^2+^ response. Shear-stress-induced Ca^2+^ influx is prevented in ECs when they are treated with an antisense oligonucleotide targeting their P2X4 channels. ATP was required to activate P2X4 and was provided in the form of endogenous ATP released by ECs [220]. When exposed to shear stress, endothelial cells (ECs) exhibit a dose-dependent release of adenosine triphosphate (ATP). Shear-stress-induced calcium (Ca^2+^) responses are completely prevented when ATP release is blocked by administration of angiostatin, an inhibitor of ATP synthase. This study suggests that endothelial cells (ECs) have the ability to effectively translate data on the magnitude of shear stress into changes in intracellular calcium (Ca^2+^) levels through the release of adenosine triphosphate (ATP) and subsequent activation of the P2X4 receptor [221].

#### 5.1.2. Tyrosine Kinase Receptors

Application of shear stress induces activation of tyrosine kinase receptors, including VEGFR2 and the angiopoietin receptor known as Tie-2. The observed activation is thought to be ligand-independent, as it is manifested in the absence of stimulation by VEGF or angiopoietin [222].

#### 5.1.3. G-Protein Coupled Receptors

G-protein coupled receptors (GPCRs) may be involved in the transduction of shear stress signals. Shear stress activation of bradykinin B2 GPCRs is demonstrated with real-time molecular imaging. This activation is facilitated by a conformational shift visualized by fluorescence resonance energy transfer in a single endothelial cell [223,224]. Activation of isolated G-proteins reconstituted into liposomes by shear stress is observed, suggesting that G-proteins alone may function as a significant mechanotransducer independent of receptor proteins [208].

#### 5.1.4. Primary Cilia

In human aortic ECs, HUVECs, and embryonic ECs, primary cilia with a rod-like, immobile structure have been shown to protrude from the apical cell membranes. Recent research shows that primary cilia are a mediator in the process by which ECs sense and respond to shear stress [225]. Because primary cilia are structurally similar to microtubules in the cytoskeleton, it is hypothesized that they may be bent by fluid flow to transmit shear stress signals into the cell. Ca^2+^ entry through ion channels may be initiated by the bending of primary cilia. Recent studies have shown that EC cilia contain the 11-transmembrane polycystin-1 protein and the TRP channel superfamily member polycystin-2, both of which function together to sense shear stress. Neither NO production nor transduction of shear stress into changes in intracellular Ca^2+^ concentration occurs in ECs lacking polycystin-1 or polycystin-2 [226].

#### 5.1.5. Glycocalyx

The glycocalyx is a layer of polymers attached to the EC membrane that covers the surface of the cell [227]. Because of its location between the cell membrane and the blood, the glycocalyx has been considered as a possible indicator of shear stress [41,228]. Flow-induced NO production in isolated canine femoral arteries is greatly reduced when hyaluronic acid glycosaminoglycans are degraded with hyaluronidase. Similarly, NO production in response to shear stress is completely abolished when heparan sulfate is removed with heparinase.

There are two hypotheses that attempt to explain the role of the EC glycocalyx in shear stress mechanotransduction. In the absence of flow, heparan sulfate proteoglycan appears as a random coil, but in the presence of flow, it unwinds into a filamentary structure [229]. Na^+^ ion binding increases with this conformational shift, suggesting that Na^+^ binding may initiate signal transduction. The glycocalyx core protein associates with the actin cytoskeleton and intracellular signaling molecules, which may transmit shear stress to the interior of the cell in addition to regulating the native concentration gradient and transporting growth factors, amino acids, and ions [208].

Shear stress activates multiple signaling pathways through a variety of membrane molecules and cellular microdomains, such as G-protein-coupled receptors, the cytoskeleton, ion channels, primary cilia, tyrosine kinase receptors, and the gylcocalyx, as shown in several studies. However, the mechanisms underlying shear stress mechanotransduction remain poorly understood [208].

### 5.2. Cytoskeletal Rearrangements

Integrins are transmembrane receptors that are critical for both the transmission of mechanical signals from cell adhesion to the ECM (extracellular matrix) and the cytoskeleton to the ECM. They form focal adhesion complexes that link the ECM to the actin cytoskeleton [230]. Integrins and focal adhesion complexes require mechanical stress stimulation, such as cyclic strain and shear stress, resulting in the promotion and activation of signaling molecules [11,12,63]. As a result of this activation, signaling pathways critical for cell survival, migration, proliferation, and cytoskeletal remodeling are activated. In addition, integrins interact with cytokines, growth factors, and other surface receptors, enabling the exchange of information between mechanical and biochemical signaling pathways. Endothelial cell behavior and cellular responses to mechanical stress are influenced by this confluence of mechanical and chemical signals [231,232].

Mechanically stress-induced cytoskeletal rearrangements are the primary method by which cells respond to mechanical stimuli. The cytoskeleton, composed of microtubules, intermediate filaments, and actin, stabilizes the structure of the cell and regulates its shape and morphology. According to Sawada and Sheetz [233], some cellular functions that rely on the ongoing reorganization of the cytoskeleton include migration, adhesion, and mechanotransduction [234]. Rearrangements of actin in the cytoskeleton induced by cyclic strain and shear stress can alter cell shape and motility. Shear stress has been shown to induce the formation of fiber stress, a cluster of actin filaments attached to focal adhesions [234]. To facilitate cell contraction, stress fibers play a role in mechanical stress transfer from the extracellular to the intracellular environment [46].

Filopodia and lamellipodia, two active membrane protrusions involved in cell migration and sensing the extracellular environment, also allow endothelial cells to enlarge in response to shear stress. Endothelial cells can orient to fluid flow and appreciate these cytoskeletal rearrangements, which also facilitate cell mobility [46,235]. The control of cytoskeletal dynamics in response to mechanical stress requires a complex interplay of signaling molecules. Cdc42, Rac1, and RhoA are GTPases that belong to the Rho family and are critical for cell motility and actin dynamics. These GTPases are activated by shear stress and cyclic strain, resulting in actin polymerization, stress fiber growth, and cytoskeletal remodeling.

Cell movement and shape changes depend on cytoskeletal reorganizations. They are also critical for controlling endothelial barrier function. Adherens junctions are important for maintaining endothelial integrity and barrier function and have been shown to be stabilized and supported by shear stress. Increased cortical actin cytoskeletal structure and strengthened adherens junctions help maintain endothelial barrier integrity and prevent excessive leakage [235,236].

Pathological mechanical stress has been associated with altered cytoskeletal organization, increased endothelial permeability, and barrier failure. The actin cytoskeleton can become disorganized under prolonged or severe mechanical stress, weakening adherens junctions and compromising barrier function. Increased endothelial permeability may allow inflammatory cells and substances to cross the endothelial barrier, contributing to the development of vascular inflammation and dysfunction [235,236]. Understanding the complex relationship between cytoskeletal rearrangements and mechanical stress and endothelial barrier function is necessary to elucidate the processes underlying vascular homeostasis and disease.

Cytokines and growth factors can be induced and produced in response to mechanical stress and act as mediators and amplifiers of how cells respond to mechanical stimuli. Growth factors, including FGFs, VEGF, and TGF, can be upregulated and released in response to mechanical stress [237,238]. These growth factors stimulate specific signaling pathways involved in cell proliferation, angiogenesis, migration, and remodeling of the ECM and associated cytoskeletal components. They also promote the synthesis and release of chemokines and cytokines that attract immune cells, control inflammation, and modify endothelial cell behavior. On the other hand, cytokines and growth factors can influence how cells respond to mechanical stress. By controlling the synthesis and function of mechanosensors, cytoskeletal elements, and integrins, they can modify the receptivity of endothelial cells to mechanical stimuli [237,238,239].

**Figure 3:**
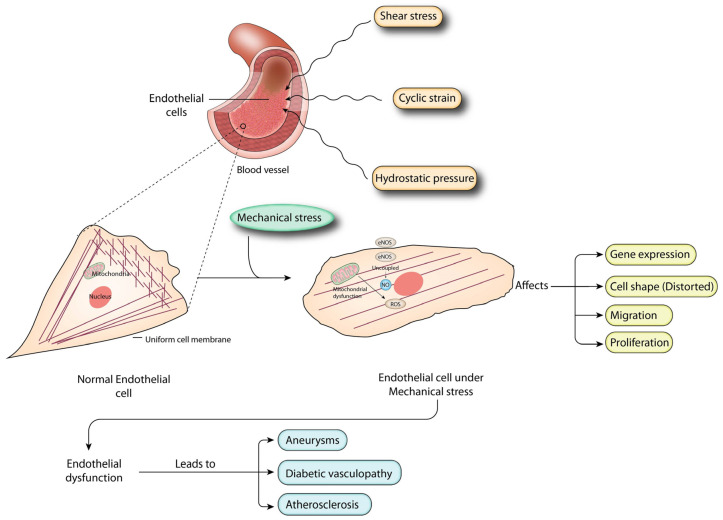
A schematic of the shear-stress-induced response in endothelial cells. Shear stress refers to the frictional force exerted by flowing blood on the endothelial cells that line blood vessels. It affects cellular signaling, gene expression, and vascular remodeling. Shear stress can also affect endothelial cell behavior, including gene expression and cell shape. Mechanical stress, including shear stress, cyclic strain, and hydrostatic pressure, can influence endothelial cell behavior by affecting gene expression, cell shape, and cell proliferation.

A schematic of the shear-stress-induced response in endothelial cells is shown in Figure 3.

## 6. Physiology and Physics of Endothelial Cells’ Response to WSS by Blood Flow

Several vascular physiological processes involve endothelial cell sensitivity to WSS, including short-term vasoreactivity, vascular remodeling, and morphogenesis [240,241].

### 6.1. Short-Term Vasoreactivity

Numerous studies over the past decades have demonstrated that shear stress induces vasodilation in an epithelium-dependent manner. On the other hand, the observation of reduced blood flow has been associated with the occurrence of vasoconstriction, which mechanistically increases WSS. In vivo experiments have shown that an increase in WSS, induced either by an increase in blood flow and wall shear rate (WSR) or by an increase in fluid viscosity, results in arterial vasodilation within a few seconds [242]. WSS induces endothelial production of vasorelaxant agonists such as prostacyclin and, most importantly, nitric oxide (NO), resulting in a rapid increase in arterial diameter [243,244]. Nitric oxide is an important vasodilator, and its production by endothelial cells in response to high WSS assists in the relaxation of vascular smooth muscle cells, resulting in minimization of vascular resistance and increased blood flow in high-WSS regions [46].

### 6.2. Shear Stress and Vascular Remodeling

In addition to the short-term vasoactive response to WSS, a sustained increase in WSS promotes long-term remodeling of the vascular wall. WSS-induced remodeling consists of several mechanisms that alter vascular homeostasis, such as endothelial cell shape, endothelial inflammation, endothelial permeability, and so on. Endothelial cells lining arterial channels in adults exhibit different morphologies depending on the direction and intensity of WSS, and their orientation aligns with the direction of flow. The flow of physiological laminar WSS induces several cellular responses, including cell alignment and elongation in the direction of flow, decreased cell proliferation, increased production of genes with anti-inflammatory properties, and suppression of inflammatory pathway expression. WSS, either above or below its set value, disrupts endothelial cell alignment, gene expression, and polarization. Its normal value alters gene expression, polarization, and EC alignment, along with stimulation of inflammation and remodeling processes [245,246].

## 7. Implications for Cardiovascular Physiology and Disease

### 7.1. Atherosclerosis and Vascular Disease

A persistent inflammatory disorder called atherosclerosis leads to plaque buildup inside the artery walls. In particular, shear stress plays an important role in the onset and development of the disease [15,33,46]. Certain areas of arteries with altered blood flow patterns experience endothelial dysfunction and have more inflammatory cell adhesion. Endothelial dysfunction leads to increased permeability, expression of adhesion molecules, and production of pro-inflammatory cytokines. This promotes the invasion of monocytes, which later mature into macrophages and consume modified LDL particles to produce foam cells. Foam cells promote the development of fatty streaks. Plaque support is provided by a fibrous cap formed by smooth muscle cells that migrate into the intima [98,247].

The extracellular matrix may deteriorate, the fibrous cap may weaken, and the plaque may become unstable. This can lead to thrombosis, plaque rupture, and major cardiovascular events, including myocardial infarction or stroke. The pathogenesis of atherosclerosis is influenced by mechanical stress, inflammation, impaired endothelial function, and plaque formation [46,48].

### 7.2. Hemodynamic Forces and Vascular Development

The modification of the vasculature by hemodynamic forces during embryonic development is influenced by endothelial cell alignment, vascular morphogenesis, and the formation of circulatory networks [248]. Shear stress significantly controls vessel width, branching, alignment, and endothelial cell behavior [249]. Shear stress gradients ensure proper delivery of nutrients and oxygen [250].

Likewise, hemodynamic stresses affect gene expression, influencing the formation of smooth muscle cells, arterial and venous identities, and specialized vascular structures [40]. Cardiovascular problems can be caused by changes in these pressures, emphasizing the need to understand their function in order to develop new treatments for vascular development [251].

Hemodynamic forces shape the vasculature throughout embryonic development to ensure proper vascular morphogenesis and function. The production of specific drugs to prevent and cure cardiovascular disease depends on our understanding of how mechanical stress affects vascular pathophysiology and development.

### 7.3. Endothelial Dysfunction in Hypertension

According to Wang et al. [252], mechanical-stress-induced endothelial dysfunction contributes significantly to the progression and development of hypertension. Endothelial cells are subjected to continuous mechanical stress due to chronic exposure to high blood pressure, which impairs their regular physiological functions [253].

In hypertension-related endothelial dysfunction, NO bioavailability is reduced, oxidative stress is increased, inflammatory processes are triggered, and the endothelial barrier function is impaired. According to Liu et al. [239] and Gallo et al. [254], these changes lead to vasoconstriction, increased vascular tone, and decreased vasodilation, contributing to the maintenance of hypertension.

The renin–angiotensin–aldosterone system is activated, prostacyclin and endothelin-1 are dysregulated, and pro-inflammatory cytokines are upregulated by mechanical stress, impairing the endothelial function, and leading to hypertension [254,255]. Understanding these pathways is necessary to develop specific treatment plans to restore the endothelial function and control hypertension.

### 7.4. Mechanotransduction in Vascular Remodeling

Mechanical stress has a significant impact on vascular remodeling, a process that involves changes in vascular structure and content. Vascular remodeling includes physiological processes such as angiogenesis and wound healing, and pathological conditions such as restenosis, atherosclerosis, and aneurysm formation. In endothelial and vascular smooth muscle cells, mechanical stress activates signaling pathways that regulate cell proliferation, migration, and matrix production and degradation. These processes affect ECM composition, wall thickness, and vessel diameter [24,33,37].

Abnormal vascular remodeling can occur under conditions of continuous mechanical stress. This can lead to neointimal hyperplasia, atherosclerotic plaque formation, and adverse arterial stiffness. Understanding the mechanotransduction processes underlying vascular remodeling will allow the development of treatments to prevent or correct pathological vascular remodeling [256,257].

## 8. Conclusions

The aorta is the largest blood vessel in the human body, carrying oxygenated blood to all organs and tissues. Abdominal aortic aneurysms (AAAs) are serious and potentially fatal vascular diseases that disproportionately affect men over the age of 65. Several pathogenic mechanisms are known to contribute to the formation of an abdominal aortic aneurysm (AAA), including vascular smooth muscle cell (VSMC) death, oxidative stress, inflammatory and immune responses, and remodeling of the vascular extracellular matrix. Remodeling of the extracellular matrix, which is primarily characterized by collagen and elastin degradation, is critical for vascular stability and the control of many vascular cell behaviors. Alterations in the extracellular matrix profile have been associated with both a larger aorta and problems with normal body function.

To study the effects of mechanical stress on endothelial cells in a controlled environment, in vitro models must be developed. By exposing endothelial cells to dynamic mechanical stresses, complex models can be developed that better reflect the complex in vivo microenvironment. It is possible to gain a better understanding of cellular responses and perform high-throughput screening for potential therapeutic therapies [258,259]. The development of high-throughput screening devices capable of systematically examining the effects of different mechanical stressors and their combinations on endothelial cells would greatly aid mechanobiology. This may facilitate the identification of important mechanosensors, signaling pathways, and potential therapeutic targets [260,261].

The integration of multi-omics techniques, including proteomics, transcriptomics, epigenomics, and genomics, may provide a thorough understanding of the molecular changes that occur in endothelial cells in response to mechanical stress. Using a multidisciplinary approach, it may be possible to identify novel mechanotransduction processes, potential biomarkers, and therapeutic targets for cardiovascular disease [262].

Understanding the underlying molecular processes and signaling pathways allows the development of new therapies and diagnostics for endothelial dysfunction and vascular remodeling. Clinical trials and extensive research are needed to demonstrate the efficacy and safety of these methods. Understanding how endothelial cells and cardiovascular health are affected by mechanical stress provides insight into cardiovascular problems and potential techniques to improve the endothelial function and treat diseases related to vascular remodeling.

In situ and in vitro research has been instrumental in discovering the biological responses and signaling pathways involved. To better understand the complexity of mechanotransduction and to contribute to the development of new treatments for cardiovascular diseases, further research using state-of-the-art models and technologies is needed.

## Data Availability

The data presented in this study are available on request from the corresponding author.

## Supplementary Materials

The following supporting information can be downloaded at: https://www.mdpi.com/article/10.3390/ijms242216518/s1.

## References

[1] Humphrey J.D., Dufresne E.R., Schwartz M.A. (2014). Mechanotransduction and extracellular matrix homeostasis. Nat. Rev. Mol. Cell Biol..

[2] Haga J.H., Li Y.-S.J., Chien S. (2007). Molecular basis of the effects of mechanical stretch on vascular smooth muscle cells. J. Biomech..

[3] Li M., Qian M., Kyler K., Xu J. (2018). Endothelial–vascular smooth muscle cells interactions in atherosclerosis. Front. Cardiovasc. Med..

[4] Yamashiro Y., Yanagisawa H. (2020). The molecular mechanism of mechanotransduction in vascular homeostasis and disease. Clin. Sci..

[5] Peng Z., Shu B., Zhang Y., Wang M. (2019). Endothelial response to pathophysiological stress. Arterioscler. Thromb. Vasc. Biol..

[6] Sandoo A., Van Zanten J.J.V., Metsios G.S., Carroll D., Kitas G.D. (2010). The endothelium and its role in regulating vascular tone. Open Cardiovasc. Med. J..

[7] Osawa M., Masuda M., Kusano K., Fujiwara K. (2002). Evidence for a role of platelet endothelial cell adhesion molecule-1 in endothelial cell mechanosignal transduction: Is it a mechanoresponsive molecule?. J. Cell Biol..

[8] Xu S., Ha C.H., Wang W., Xu X., Yin M., Jin F.Q., Mastrangelo M., Koroleva M., Fujiwara K., Jin Z.G. (2016). PECAM1 regulates flow-mediated Gab1 tyrosine phosphorylation and signaling. Cell. Signal..

[9] Fang Y., Wu D., Birukov K.G. (2019). Mechanosensing and mechanoregulation of endothelial cell functions. Compr. Physiol..

[10] Bartosch A.M.W., Mathews R., Mahmoud M.M., Cancel L.M., Haq Z.S., Tarbell J.M. (2021). Heparan sulfate proteoglycan glypican-1 and PECAM-1 cooperate in shear-induced endothelial nitric oxide production. Sci. Rep..

[11] Katoh K., Masuda M., Kano Y., Jinguji Y., Fujiwara K. (1995). Focal adhesion proteins associated with apical stress fibers of human fibroblasts. Cell Motil. Cytoskelet..

[12] Kano Y., Katoh K., Fujiwara K. (2000). Lateral zone of cell-cell adhesion as the major fluid shear stress-related signal transduction site. Circ. Res..

[13] Dejana E. (2004). Endothelial cell-cell junctions: Happy together. Nat. Rev. Mol. Cell Biol..

[14] Krüger-Genge A., Blocki A., Franke R.-P., Jung F. (2019). Vascular endothelial cell biology: An update. Int. J. Mol. Sci..

[15] Chiu J.-J., Chien S. (2011). Effects of disturbed flow on vascular endothelium: Pathophysiological basis and clinical perspectives. Physiol. Rev..

[16] Davis M.J., Earley S., Li Y.-S., Chien S. (2023). Vascular mechanotransduction. Physiol. Rev..

[17] Samijo S.K., Willigers J.M., Barkhuysen R., Kitslaar P.J., Reneman R.S., Brands P.J., Hoeks A.P. (1998). Wall shear stress in the human common carotid artery as function of age and gender. Cardiovasc. Res..

[18] Sakariassen K.S., Orning L., Turitto V.T. (2015). The impact of blood shear rate on arterial thrombus formation. Future Sci. OA.

[19] Kim D., Bresette C., Liu Z., Ku D.N. (2019). Occlusive thrombosis in arteries. APL Bioeng..

[20] Gnasso A., Carallo C., Irace C., Spagnuolo V., De Novara G., Mattioli P.L., Pujia A. (1996). Association between intima-media thickness and wall shear stress in common carotid arteries in healthy male subjects. Circulation.

[21] Wyk S., Wittberg L.P., Bulusu K.V., Fuchs L., Plesniak M.W. (2015). Non-Newtonian perspectives on pulsatile blood-analog flows in a 180° curved artery model. Phys. Fluids.

[22] Liu H., Lan L., Abrigo J., Ip H.L., Soo Y., Zheng D., Wong K.S., Wang D., Shi L., Leung T.W. (2021). Comparison of Newtonian and Non-newtonian Fluid Models in Blood Flow Simulation in Patients With Intracranial Arterial Stenosis. Front. Physiol..

[23] Papaioannou T.G., Stefanadis C. (2005). Vascular wall shear stress: Basic principles and methods. Hell. J. Cardiol..

[24] Hoefer I.E., den Adel B., Daemen M.J. (2013). Biomechanical factors as triggers of vascular growth. Cardiovasc. Res..

[25] Camasao D.B., Mantovani D. (2021). The mechanical characterization of blood vessels and their substitutes in the continuous quest for physiological-relevant performances. A critical review. Mater. Today Bio.

[26] Wang C., Guo X., Kassab G.S. (2009). A new observation on the stress distribution in the coronary artery wall. J. Biomech. Eng..

[27] Pries A.R., Secomb T.W. (2005). Control of blood vessel structure: Insights from theoretical models. Am. J. Physiol. Heart Circ. Physiol..

[28] Pries A.R., Reglin B., Secomb T.W. (2005). Remodeling of blood vessels: Responses of diameter and wall thickness to hemodynamic and metabolic stimuli. Hypertension.

[29] Doriot P.A., Dorsaz P.A. (2003). Is axial wall stress compressive in certain arteries?. Med. Phys..

[30] Liu J., Khalil R.A. (2017). Matrix Metalloproteinase Inhibitors as Investigational and Therapeutic Tools in Unrestrained Tissue Remodeling and Pathological Disorders. Prog. Mol. Biol. Transl. Sci..

[31] Shimoda M. (2019). Extracellular vesicle-associated MMPs: A modulator of the tissue microenvironment. Adv. Clin. Chem..

[32] Wang X., Khalil R.A. (2018). Matrix Metalloproteinases, Vascular Remodeling, and Vascular Disease. Adv. Pharmacol..

[33] Wang X., Shen Y., Shang M., Liu X., Munn L.L. (2023). Endothelial mechanobiology in atherosclerosis. Cardiovasc. Res..

[34] Mishani S., Belhoul-Fakir H., Lagat C., Jansen S., Evans B., Lawrence-Brown M. (2021). Stress distribution in the walls of major arteries: Implications for atherogenesis. Quant. Imaging Med. Surg..

[35] Cui X., Tong J., Yau J., Bajpai A., Yang J., Peng Y., Singh M., Qian W., Ma X., Chen W. (2020). Mechanical Forces Regulate Asymmetric Vascular Cell Alignment. Biophys. J..

[36] Gimbrone M.A., Anderson K.R., Topper J.N., Langille B.L., Clowes A.W., Bercel S., Davies M.G., Stenmark K.R., Frid M.G., Weiser-Evans M.C. (1999). Special communicationthe critical role of mechanical forces in blood vessel development, physiology and pathology. J. Vasc. Surg..

[37] Renna N.F., de Las Heras N., Miatello R.M. (2013). Pathophysiology of vascular remodeling in hypertension. Int. J. Hypertens..

[38] Martinez-Quinones P., McCarthy C.G., Watts S.W., Klee N.S., Komic A., Calmasini F.B., Priviero F., Warner A., Chenghao Y., Wenceslau C.F. (2018). Hypertension Induced Morphological and Physiological Changes in Cells of the Arterial Wall. Am. J. Hypertens..

[39] Lehoux S., Tedgui A. (1998). Signal transduction of mechanical stresses in the vascular wall. Hypertension.

[40] Chien S. (2007). Mechanotransduction and endothelial cell homeostasis: The wisdom of the cell. Am. J. Physiol.-Heart Circ. Physiol..

[41] Tarbell J.M., Pahakis M. (2006). Mechanotransduction and the glycocalyx. J. Intern. Med..

[42] Cunningham K.S., Gotlieb A.I. (2005). The role of shear stress in the pathogenesis of atherosclerosis. Lab. Investig..

[43] Khan A.W., Paneni F., Jandeleit-Dahm K.A.M. (2021). Cell-specific epigenetic changes in atherosclerosis. Clin. Sci..

[44] Pries A.R., Secomb T.W., Gaehtgens P. (2000). The endothelial surface layer. Pflügers Arch..

[45] Shyy J.Y.-J., Chien S. (2002). Role of integrins in endothelial mechanosensing of shear stress. Circ. Res..

[46] Davies P.F. (2009). Hemodynamic shear stress and the endothelium in cardiovascular pathophysiology. Nat. Clin. Pract. Cardiovasc. Med..

[47] Malek A.M., Izumo S. (1996). Mechanism of endothelial cell shape change and cytoskeletal remodeling in response to fluid shear stress. J. Cell Sci..

[48] Hahn C., Schwartz M.A. (2009). Mechanotransduction in vascular physiology and atherogenesis. Nat. Rev. Mol. Cell Biol..

[49] Rubanyi G.M., Romero J.C., Vanhoutte P.M. (1986). Flow-induced release of endothelium-derived relaxing factor. Am. J. Physiol.-Heart Circ. Physiol..

[50] Boo Y.C., Jo H. (2003). Flow-dependent regulation of endothelial nitric oxide synthase: Role of protein kinases. Am. J. Physiol.-Cell Physiol..

[51] Ohno M., Gibbons G.H., Dzau V.J., Cooke J.P. (1993). Shear stress elevates endothelial cGMP. Role of a potassium channel and G protein coupling. Circulation.

[52] Corson M.A., James N.L., Latta S.E., Nerem R.M., Berk B.C., Harrison D.G. (1996). Phosphorylation of endothelial nitric oxide synthase in response to fluid shear stress. Circ. Res..

[53] Fleming I., Bauersachs J., Fisslthaler B., Busse R. (1998). Ca^2+^-independent activation of the endothelial nitric oxide synthase in response to tyrosine phosphatase inhibitors and fluid shear stress. Circ. Res..

[54] Uematsu M., Ohara Y., Navas J.P., Nishida K., Murphy T., Alexander R.W., Nerem R.M., Harrison D.G. (1995). Regulation of endothelial cell nitric oxide synthase mRNA expression by shear stress. Am. J. Physiol.-Cell Physiol..

[55] Weber M., Hagedorn C.H., Harrison D.G., Searles C.D. (2005). Laminar shear stress and 3′ polyadenylation of eNOS mRNA. Circ. Res..

[56] Chun T.-H., Itoh H., Ogawa Y., Tamura N., Takaya K., Igaki T., Yamashita J., Doi K., Inoue M., Masatsugu K. (1997). Shear stress augments expression of C-type natriuretic peptide and adrenomedullin. Hypertension.

[57] Rieder M., Carmona R., Krieger J., Pritchard Jr K., Greene A. (1997). Suppression of angiotensin-converting enzyme expression and activity by shear stress. Circ. Res..

[58] Katoh K., Kano Y., Fujiwara K. (2000). Isolation and in vitro contraction of stress fibers. Methods Enzym..

[59] Masuda M., Osawa M., Shigematsu H., Harada N., Fujiwara K. (1997). Platelet endothelial cell adhesion molecule-1 is a major SH-PTP2 binding protein in vascular endothelial cells. FEBS Lett..

[60] Osawa M., Masuda M., Harada N., Lopes R.B., Fujiwara K. (1997). Tyrosine phosphorylation of platelet endothelial cell adhesion molecule-1 (PECAM-1, CD31) in mechanically stimulated vascular endothelial cells. Eur. J. Cell Biol..

[61] Noel J., Wang H., Hong N., Tao J.Q., Yu K., Sorokina E.M., Debolt K., Heayn M., Rizzo V., Delisser H. (2013). PECAM-1 and caveolae form the mechanosensing complex necessary for NOX2 activation and angiogenic signaling with stopped flow in pulmonary endothelium. Am. J. Physiol. Lung Cell Mol. Physiol..

[62] Gliemann L., Rytter N., Piil P., Nilton J., Lind T., Nyberg M., Cocks M., Hellsten Y. (2018). The Endothelial Mechanotransduction Protein Platelet Endothelial Cell Adhesion Molecule-1 Is Influenced by Aging and Exercise Training in Human Skeletal Muscle. Front. Physiol..

[63] Kano Y., Katoh K., Masuda M., Fujiwara K. (1996). Macromolecular composition of stress fiber-plasma membrane attachment sites in endothelial cells in situ. Circ. Res..

[64] Katoh K., Kano Y., Ookawara S. (2007). Morphological differences between guinea pig aortic and venous endothelial cells in situ. Cell Biol. Int..

[65] Hudlicka O. (1993). Physical forces and angiogenesis. Mechanorecept. Vasc. Wall.

[66] Kamiya A., Togawa T. (1980). Adaptive regulation of wall shear stress to flow change in the canine carotid artery. Am. J. Physiol.-Heart Circ. Physiol..

[67] Wanhainen A., Hultgren R., Linné A. (2017). Outcome of the swedish nationwide abdominal aortic aneurysm screening program. J. Vasc. Surg..

[68] Quintana R.A., Taylor W.R. (2019). Cellular mechanisms of aortic aneurysm formation. Circ. Res..

[69] Halper J. (2018). Basic components of vascular connective tissue and extracellular matrix. Adv. Pharmacol..

[70] Theocharis A.D., Skandalis S.S., Gialeli C., Karamanos N.K. (2016). Extracellular matrix structure. Adv. Drug Deliv. Rev..

[71] Dobrin P., Mrkvicka R. (1994). Failure of elastin or collagen as possible critical connective tissue alterations underlying aneurysmal dilatation. Cardiovasc. Surg..

[72] Didangelos A., Yin X., Mandal K., Saje A., Smith A., Xu Q., Jahangiri M., Mayr M. (2011). Extracellular matrix composition and remodeling in human abdominal aortic aneurysms: A proteomics approach. Mol. Cell. Proteom..

[73] Tanios F., Gee M., Pelisek J., Kehl S., Biehler J., Grabher-Meier V., Wall W., Eckstein H.-H., Reeps C. (2015). Interaction of biomechanics with extracellular matrix components in abdominal aortic aneurysm wall. Eur. J. Vasc. Endovasc. Surg..

[74] Incalza M.A., D’Oria R., Natalicchio A., Perrini S., Laviola L., Giorgino F. (2018). Oxidative stress and reactive oxygen species in endothelial dysfunction associated with cardiovascular and metabolic diseases. Vasc. Pharmacol..

[75] Montezano A.C., Touyz R.M. (2012). Reactive oxygen species and endothelial function--role of nitric oxide synthase uncoupling and Nox family nicotinamide adenine dinucleotide phosphate oxidases. Basic. Clin. Pharmacol. Toxicol..

[76] Guzik B., Sagan A., Ludew D., Mrowiecki W., Chwała M., Bujak-Gizycka B., Filip G., Grudzien G., Kapelak B., Zmudka K. (2013). Mechanisms of oxidative stress in human aortic aneurysms—Association with clinical risk factors for atherosclerosis and disease severity. Int. J. Cardiol..

[77] DiMusto P.D., Lu G., Ghosh A., Roelofs K.J., Sadiq O., McEvoy B., Su G., Laser A., Bhamidipati C.M., Ailawadi G. (2012). Increased JNK in males compared with females in a rodent model of abdominal aortic aneurysm. J. Surg. Res..

[78] Yoshimura K., Aoki H., Ikeda Y., Fujii K., Akiyama N., Furutani A., Hoshii Y., Tanaka N., Ricci R., Ishihara T. (2005). Regression of abdominal aortic aneurysm by inhibition of c-Jun N-terminal kinase. Nat. Med..

[79] Lemaître V., Dabo A.J., D’Armiento J. (2011). Cigarette smoke components induce matrix metalloproteinase-1 in aortic endothelial cells through inhibition of mTOR signaling. Toxicol. Sci..

[80] Zhang C., Zhang M.-X., Shen Y.H., Burks J.K., Zhang Y., Wang J., LeMaire S.A., Yoshimura K., Aoki H., Coselli J.S. (2007). TNF-α suppresses prolyl-4-hydroxylase α1 expression via the ASK1–JNK–NonO pathway. Arterioscler. Thromb. Vasc. Biol..

[81] Vincenti M.P., Brinckerhoff C.E. (2007). Signal transduction and cell-type specific regulation of matrix metalloproteinase gene expression: Can MMPs be good for you?. J. Cell. Physiol..

[82] Miyake T., Aoki M., Nakashima H., Kawasaki T., Oishi M., Kataoka K., Tanemoto K., Ogihara T., Kaneda Y., Morishita R. (2006). Prevention of abdominal aortic aneurysms by simultaneous inhibition of NFκB and ets using chimeric decoy oligonucleotides in a rabbit model. Gene Ther..

[83] Miyake T., Aoki M., Masaki H., Kawasaki T., Oishi M., Kataoka K., Ogihara T., Kaneda Y., Morishita R. (2007). Regression of abdominal aortic aneurysms by simultaneous inhibition of nuclear factor κB and ets in a rabbit model. Circ. Res..

[84] Li Z., Kong W. (2020). Cellular signaling in abdominal aortic aneurysm. Cell. Signal..

[85] Biros E., Walker P.J., Nataatmadja M., West M., Golledge J. (2012). Downregulation of transforming growth factor, beta receptor 2 and Notch signaling pathway in human abdominal aortic aneurysm. Atherosclerosis.

[86] Dai J., Losy F., Guinault A.-M., Pages C., Anegon I., Desgranges P., Becquemin J.-P., Allaire E. (2005). Overexpression of transforming growth factor-β1 stabilizes already-formed aortic aneurysms: A first approach to induction of functional healing by endovascular gene therapy. Circulation.

[87] Wang Y., Ait-Oufella H., Herbin O., Bonnin P., Ramkhelawon B., Taleb S., Huang J., Offenstadt G., Combadière C., Rénia L. (2010). TGF-β activity protects against inflammatory aortic aneurysm progression and complications in angiotensin II–infused mice. J. Clin. Investig..

[88] Zhang P., Hou S., Chen J., Zhang J., Lin F., Ju R., Cheng X., Ma X., Song Y., Zhang Y. (2016). Smad4 deficiency in smooth muscle cells initiates the formation of aortic aneurysm. Circ. Res..

[89] Tan C.K., Tan E.H., Luo B., Huang C.L., Loo J.S., Choong C., Tan N.S. (2013). SMAD3 deficiency promotes inflammatory aortic aneurysms in angiotensin II–infused mice via activation of iNOS. J. Am. Heart Assoc..

[90] Womersley J.R. (1955). Method for the calculation of velocity, rate of flow and viscous drag in arteries when the pressure gradient is known. J. Physiol..

[91] Womersley J.R. (1957). Oscillatory flow in arteries: The constrained elastic tube as a model of arterial flow and pulse transmission. Phys. Med. Biol..

[92] Womersley J.R. (1958). Oscillatory flow in arteries. III. Flow and pulse-velocity formulae for a liquid whose viscosity varies with frequency. Phys. Med. Biol..

[93] Saqr K.M., Rashad S., Tupin S., Niizuma K., Hassan T., Tominaga T., Ohta M. (2020). What does computational fluid dynamics tell us about intracranial aneurysms? A meta-analysis and critical review. J. Cereb. Blood Flow. Metab..

[94] Saqr K.M., Tupin S., Rashad S., Endo T., Niizuma K., Tominaga T., Ohta M. (2020). Physiologic blood flow is turbulent. Sci. Rep..

[95] Lee S.W., Antiga L., Spence J.D., Steinman D.A. (2008). Geometry of the carotid bifurcation predicts its exposure to disturbed flow. Stroke.

[96] Toole J.F., Castaldo J.E. (1994). Accurate measurement of carotid stenosis. Chaos in methodology. J. Neuroimaging.

[97] Nam D., Ni C.W., Rezvan A., Suo J., Budzyn K., Llanos A., Harrison D., Giddens D., Jo H. (2009). Partial carotid ligation is a model of acutely induced disturbed flow, leading to rapid endothelial dysfunction and atherosclerosis. Am. J. Physiol. Heart Circ. Physiol..

[98] Chatzizisis Y.S., Coskun A.U., Jonas M., Edelman E.R., Feldman C.L., Stone P.H. (2007). Role of endothelial shear stress in the natural history of coronary atherosclerosis and vascular remodeling: Molecular, cellular, and vascular behavior. J. Am. Coll. Cardiol..

[99] Ai L., Yu H., Dai W., Hale S.L., Kloner R.A., Hsiai T.K. (2009). Real-time intravascular shear stress in the rabbit abdominal aorta. IEEE Trans. Biomed. Eng..

[100] Ross R. (1999). Atherosclerosis—An inflammatory disease. N. Engl. J. Med..

[101] Niebauer J., Cooke J.P. (1996). Cardiovascular effects of exercise: Role of endothelial shear stress. J. Am. Coll. Cardiol..

[102] Hwang J., Rouhanizadeh M., Hamilton R.T., Lin T.C., Eiserich J.P., Hodis H.N., Hsiai T.K. (2006). 17β-Estradiol reverses shear-stress-mediated low density lipoprotein modifications. Free Radic. Biol. Med..

[103] Raghavan M.L., Vorp D.A., Federle M.P., Makaroun M.S., Webster M.W. (2000). Wall stress distribution on three-dimensionally reconstructed models of human abdominal aortic aneurysm. J. Vasc. Surg..

[104] Stone P.H., Coskun A.U., Kinlay S., Clark M.E., Sonka M., Wahle A., Ilegbusi O.J., Yeghiazarians Y., Popma J.J., Orav J. (2003). Effect of endothelial shear stress on the progression of coronary artery disease, vascular remodeling, and in-stent restenosis in humans: In vivo 6-month follow-up study. Circulation.

[105] Zhou X., Yin L., Xu L., Liang F. (2020). Non-periodicity of blood flow and its influence on wall shear stress in the carotid artery bifurcation: An in vivo measurement-based computational study. J. Biomech..

[106] Li X., Liu X., Li X., Xu L., Chen X., Liang F. (2019). Tortuosity of the superficial femoral artery and its influence on blood flow patterns and risk of atherosclerosis. Biomech. Model. Mechanobiol..

[107] Hoi Y., Wasserman B.A., Lakatta E.G., Steinman D.A. (2010). Carotid bifurcation hemodynamics in older adults: Effect of measured versus assumed flow waveform. J. Biomech. Eng..

[108] Katoh K., Noda Y. (2012). Distributuion of cytoskeletal components in endothelial cells in the guinea pig renal artery. Int. J. Cell Biol..

[109] Taylor C.A., Figueroa C. (2009). Patient-specific modeling of cardiovascular mechanics. Annu. Rev. Biomed. Eng..

[110] Khader S.M.A., Azriff A., Johny C., Pai R., Zuber M., Ahmad K.A., Ahmad Z. (2018). Haemodynamics behaviour in normal and stenosed renal artery using computational fluid dynamics. J. Adv. Res. Fluid. Mech. Therm. Sci..

[111] Brozovich F., Nicholson C., Degen C., Gao Y.Z., Aggarwal M., Morgan K. (2016). Mechanisms of vascular smooth muscle contraction and the basis for pharmacologic treatment of smooth muscle disorders. Pharmacol. Rev..

[112] Wang Y., Baeyens N., Corti F., Tanaka K., Fang J.S., Zhang J., Jin Y., Coon B., Hirschi K.K., Schwartz M.A. (2016). Syndecan 4 controls lymphatic vasculature remodeling during mouse embryonic development. Development.

[113] Zhang W., Huang G., Xu F. (2020). Engineering biomaterials and approaches for mechanical stretching of cells in three dimensions. Front. Bioeng. Biotechnol..

[114] Balachandran K., Sucosky P., Jo H., Yoganathan A.P. (2009). Elevated cyclic stretch alters matrix remodeling in aortic valve cusps: Implications for degenerative aortic valve disease. Am. J. Physiol.-Heart Circ. Physiol..

[115] Fisher A.B., Chien S., Barakat A.I., Nerem R.M. (2001). Endothelial cellular response to altered shear stress. Am. J. Physiol. Lung Cell Mol. Physiol..

[116] Tesauro M., Cardillo C. (2011). Obesity, blood vessels and metabolic syndrome. Acta Physiol..

[117] Michiels C. (2003). Endothelial cell functions. J. Cell Physiol..

[118] Gori T. (2018). Endothelial Function: A Short Guide for the Interventional Cardiologist. Int. J. Mol. Sci..

[119] Tansey E.A., Montgomery L.E., Quinn J.G., Roe S.M., Johnson C.D. (2019). Understanding basic vein physiology and venous blood pressure through simple physical assessments. Adv. Physiol. Educ..

[120] Vozzi F., Bianchi F., Ahluwalia A., Domenici C. (2014). Hydrostatic pressure and shear stress affect endothelin-1 and nitric oxide release by endothelial cells in bioreactors. Biotechnol. J..

[121] Coughlin M.F., Sohn D.D., Schmid-Schönbein G.W. (2008). Recoil and stiffening by adherent leukocytes in response to fluid shear. Biophys. J..

[122] Müller-Marschhausen K., Waschke J., Drenckhahn D. (2008). Physiological hydrostatic pressure protects endothelial monolayer integrity. Am. J. Physiol. Cell Physiol..

[123] Yoshino D., Funamoto K., Sato K., Kenry, Sato M., Lim C.T. (2020). Hydrostatic pressure promotes endothelial tube formation through aquaporin 1 and Ras-ERK signaling. Commun. Biol..

[124] Mammoto T., Hunyenyiwa T., Kyi P., Hendee K., Matus K., Rao S., Lee S.H., Tabima D.M., Chesler N.C., Mammoto A. (2022). Hydrostatic Pressure Controls Angiogenesis through Endothelial YAP1 During Lung Regeneration. Front. Bioeng. Biotechnol..

[125] Komarova Y.A., Kruse K., Mehta D., Malik A.B. (2017). Protein Interactions at Endothelial Junctions and Signaling Mechanisms Regulating Endothelial Permeability. Circ. Res..

[126] Seccia T.M., Rigato M., Ravarotto V., Calò L.A. (2020). ROCK (RhoA/Rho Kinase) in Cardiovascular–Renal Pathophysiology: A Review of New Advancements. J. Clin. Med..

[127] Lawrence T. (2009). The nuclear factor NF-κB pathway in inflammation. Cold Spring Harb. Perspect. Biol..

[128] Liu T., Zhang L., Joo D., Sun S.C. (2017). NF-κB signaling in inflammation. Signal Transduct. Target. Ther..

[129] Tousoulis D., Briasoulis A., Papageorgiou N., Tsioufis C., Tsiamis E., Toutouzas K., Stefanadis C. (2011). Oxidative stress and endothelial function: Therapeutic interventions. Recent Pat. Cardiovasc. Drug Discov. Discontin..

[130] Gimbrone M.A., Cotran R.S., Folkman J. (1974). Human vascular endothelial cells in culture. Growth and DNA synthesis. J. Cell Biol..

[131] Booyse F.M., Sedlak B.J., Rafelson M.E. (1975). Culture of arterial endothelial cells: Characterization and growth of bovine aortic cells. Thromb. Diath. Haemorrh..

[132] Meng F., Cheng H., Qian J., Dai X., Huang Y., Fan Y. (2022). In vitro fluidic systems: Applying shear stress on endothelial cells. Med. Nov. Technol. Devices.

[133] Chen J., Zhou Y., Liu S., Li C. (2020). Biomechanical signal communication in vascular smooth muscle cells. J. Cell Commun. Signal..

[134] Haubner F., Leyh M., Ohmann E., Pohl F., Prantl L., Gassner H.G. (2013). Effects of external radiation in a co-culture model of endothelial cells and adipose-derived stem cells. Radiat. Oncol..

[135] Swaminathan S., Cranston A.N., Clyne A.M. (2019). A Three-Dimensional In Vitro Coculture Model to Quantify Breast Epithelial Cell Adhesion to Endothelial Cells. Tissue Eng. Part C Methods.

[136] Kanczler J.M., Wells J.A., Oreffo R.O.C. (2021). Endothelial Cells: Co-culture Spheroids. Methods Mol. Biol..

[137] Odell A.F., Mannion A.J. (2022). In Vitro Co-culture of Fibroblast and Endothelial Cells to Assess Angiogenesis. Methods Mol. Biol..

[138] Liu M., Samant S., Vasa C.H., Pedrigi R.M., Oguz U.M., Ryu S., Wei T., Anderson D.R., Agrawal D.K., Chatzizisis Y.S. (2023). Co-culture models of endothelial cells, macrophages, and vascular smooth muscle cells for the study of the natural history of atherosclerosis. PLoS ONE.

[139] Wong A.K., Llanos P., Boroda N., Rosenberg S.R., Rabbany S.Y. (2016). A Parallel-Plate Flow Chamber for Mechanical Characterization of Endothelial Cells Exposed to Laminar Shear Stress. Cell Mol. Bioeng..

[140] t Hart D.C., van der Vlag J., Nijenhuis T. (2021). Laminar flow substantially affects the morphology and functional phenotype of glomerular endothelial cells. PLoS ONE.

[141] Wakida N.M., Cruz G.M.S., Pouladian P., Berns M.W., Preece D. (2020). Fluid Shear Stress Enhances the Phagocytic Response of Astrocytes. Front. Bioeng. Biotechnol..

[142] Xanthis I., Souilhol C., Serbanovic-Canic J., Roddie H., Kalli A.C., Fragiadaki M., Wong R., Shah D.R., Askari J.A., Canham L. (2019). β1 integrin is a sensor of blood flow direction. J. Cell Sci..

[143] Keeley T.P., Siow R.C.M., Jacob R., Mann G.E. (2017). A PP2A-mediated feedback mechanism controls Ca^2+^-dependent NO synthesis under physiological oxygen. FASEB J. Off. Publ. Fed. Am. Soc. Exp. Biol..

[144] Patton J.T., Menter D.G., Benson D.M., Nicolson G.L., McIntire L.V. (1993). Computerized analysis of tumor cells flowing in a parallel plate chamber to determine their adhesion stabilization lag time. Cell Motil. Cytoskelet..

[145] Sorescu G.P., Sykes M., Weiss D., Platt M.O., Saha A., Hwang J., Boyd N., Boo Y.C., Vega J.D., Taylor W.R. (2003). Bone morphogenic protein 4 produced in endothelial cells by oscillatory shear stress stimulates an inflammatory response. J. Biol. Chem..

[146] Yoshizumi M., Kurihara H., Sugiyama T., Takaku F., Yanagisawa M., Masaki T., Yazaki Y. (1989). Hemodynamic shear stress stimulates endothelin production by cultured endothelial cells. Biochem. Biophys. Res. Commun..

[147] Ley K., Lundgren E., Berger E., Arfors K.E. (1989). Shear-dependent inhibition of granulocyte adhesion to cultured endothelium by dextran sulfate. Blood.

[148] Pearce M.J., McIntyre T.M., Prescott S.M., Zimmerman G.A., Whatley R.E. (1996). Shear stress activates cytosolic phospholipase A2 (cPLA2) and MAP kinase in human endothelial cells. Biochem. Biophys. Res. Commun..

[149] Asada H., Paszkowiak J., Teso D., Alvi K., Thorisson A., Frattini J.C., Kudo F.A., Sumpio B.E., Dardik A. (2005). Sustained orbital shear stress stimulates smooth muscle cell proliferation via the extracellular signal-regulated protein kinase 1/2 pathway. J. Vasc. Surg..

[150] Feng S., Mao S., Zhang Q., Li W., Lin J.M. (2019). Online Analysis of Drug Toxicity to Cells with Shear Stress on an Integrated Microfluidic Chip. ACS Sens..

[151] Chau L., Doran M., Cooper-White J. (2009). A novel multishear microdevice for studying cell mechanics. Lab Chip.

[152] Langhans S.A. (2018). Three-dimensional in vitro cell culture models in drug discovery and drug repositioning. Front. Pharmacol..

[153] Griffith C.M., Huang S.A., Cho C., Khare T.M., Rich M., Lee G.-h., Ligler F.S., Diekman B.O., Polacheck W.J. (2020). Microfluidics for the study of mechanotransduction. J. Phys. D Appl. Phys..

[154] Li X., Valadez A.V., Zuo P., Nie Z. (2012). Microfluidic 3D cell culture: Potential application for tissue-based bioassays. Bioanalysis.

[155] Atcha H., Davis C.T., Sullivan N.R., Smith T.D., Anis S., Dahbour W.Z., Robinson Z.R., Grosberg A., Liu W.F. (2018). A low-cost mechanical stretching device for uniaxial strain of cells: A platform for pedagogy in mechanobiology. J. Biomech. Eng..

[156] Schaffer J.L., Rizen M., L’Italien G.J., Benbrahim A., Megerman J., Gerstenfeld L.C., Gray M.L. (1994). Device for the application of a dynamic biaxially uniform and isotropic strain to a flexible cell culture membrane. J. Orthop. Res..

[157] Reinwald Y., Leonard K.H., Henstock J.R., Whiteley J.P., Osborne J.M., Waters S.L., Levesque P., El Haj A.J. (2015). Evaluation of the growth environment of a hydrostatic force bioreactor for preconditioning of tissue-engineered constructs. Tissue Eng. Part C Methods.

[158] Zvicer J., Obradovic B. (2018). Bioreactors with hydrostatic pressures imitating physiological environments in intervertebral discs. J. Tissue Eng. Regen. Med..

[159] Ohashi T., Sugaya Y., Sakamoto N., Sato M. (2007). Hydrostatic pressure influences morphology and expression of VE-cadherin of vascular endothelial cells. J. Biomech..

[160] Charbonier F.W., Zamani M., Huang N.F. (2019). Endothelial cell mechanotransduction in the dynamic vascular environment. Adv. Biosyst..

[161] Cahill P.A., Redmond E.M. (2016). Vascular endothelium—Gatekeeper of vessel health. Atherosclerosis.

[162] Adil M.S., Narayanan S.P., Somanath P.R. (2021). Cell-cell junctions: Structure and regulation in physiology and pathology. Tissue Barriers.

[163] Gulino-Debrac D. (2013). Mechanotransduction at the basis of endothelial barrier function. Tissue Barriers.

[164] Sukriti S., Tauseef M., Yazbeck P., Mehta D. (2014). Mechanisms regulating endothelial permeability. Pulm. Circ..

[165] Claesson-Welsh L., Dejana E., McDonald D.M. (2021). Permeability of the endothelial barrier: Identifying and reconciling controversies. Trends Mol. Med..

[166] Schött U., Solomon C., Fries D., Bentzer P. (2016). The endothelial glycocalyx and its disruption, protection and regeneration: A narrative review. Scand. J. Trauma Resusc. Emerg. Med..

[167] Li Y., Zheng J., Bird I.M., Magness R.R. (2004). Mechanisms of shear stress-induced endothelial nitric-oxide synthase phosphorylation and expression in ovine fetoplacental artery endothelial cells. Biol. Reprod..

[168] Sriram K., Laughlin J.G., Rangamani P., Tartakovsky D.M. (2016). Shear-induced nitric oxide production by endothelial cells. Biophys. J..

[169] Boo Y.C., Hwang J., Sykes M., Michell B.J., Kemp B.E., Lum H., Jo H. (2002). Shear stress stimulates phosphorylation of eNOS at Ser635 by a protein kinase A-dependent mechanism. Am. J. Physiol.-Heart Circ. Physiol..

[170] Kubes P., Suzuki M., Granger D.N. (1991). Nitric oxide: An endogenous modulator of leukocyte adhesion. Proc. Natl. Acad. Sci. USA.

[171] Riddell D.R., Owen J.S. (1999). Nitric oxide and platelet aggregation. Vitam. Horm..

[172] Liao J.K. (2013). Linking endothelial dysfunction with endothelial cell activation. J. Clin. Investig..

[173] Gao F., Lucke-Wold B.P., Li X., Logsdon A.F., Xu L.C., Xu S., LaPenna K.B., Wang H., Talukder M.A.H., Siedlecki C.A. (2017). Reduction of Endothelial Nitric Oxide Increases the Adhesiveness of Constitutive Endothelial Membrane ICAM-1 through Src-Mediated Phosphorylation. Front. Physiol..

[174] Pilard M., Ollivier E.L., Gourdou-Latyszenok V., Couturaud F., Lemarié C.A. (2022). Endothelial cell phenotype, a major determinant of venous thrombo-inflammation. Front. Cardiovasc. Med..

[175] Ziegler T., Silacci P., Harrison V.J., Hayoz D. (1998). Nitric oxide synthase expression in endothelial cells exposed to mechanical forces. Hypertension.

[176] Balligand J.L., Feron O., Dessy C. (2009). eNOS activation by physical forces: From short-term regulation of contraction to chronic remodeling of cardiovascular tissues. Physiol. Rev..

[177] Boycott H.E., Nguyen M.N., Vrellaku B., Gehmlich K., Robinson P. (2020). Nitric Oxide and Mechano-Electrical Transduction in Cardiomyocytes. Front. Physiol..

[178] Uchida T., Shimizu S., Yamagishi R., Tokuoka S.M., Kita Y., Honjo M., Aihara M. (2021). Mechanical stretch induces Ca^2+^ influx and extracellular release of PGE_2_ through Piezo1 activation in trabecular meshwork cells. Sci. Rep..

[179] Ignarro L.J. (1989). Endothelium-derived nitric oxide: Actions and properties. FASEB J. Off. Publ. Fed. Am. Soc. Exp. Biol..

[180] Rafikov R., Fonseca F.V., Kumar S., Pardo D., Darragh C., Elms S., Fulton D., Black S.M. (2011). eNOS activation and NO function: Structural motifs responsible for the posttranslational control of endothelial nitric oxide synthase activity. J. Endocrinol..

[181] Fleming I. (2010). Molecular mechanisms underlying the activation of eNOS. Pflug. Arch..

[182] Fleming I., Busse R. (1999). Signal transduction of eNOS activation. Cardiovasc. Res..

[183] Lundberg J.O., Weitzberg E. (2022). Nitric oxide signaling in health and disease. Cell.

[184] Gantner B.N., LaFond K.M., Bonini M.G. (2020). Nitric oxide in cellular adaptation and disease. Redox Biol..

[185] Pourbagher-Shahri A.M., Farkhondeh T., Talebi M., Kopustinskiene D.M., Samarghandian S., Bernatoniene J. (2021). An Overview of NO Signaling Pathways in Aging. Molecules.

[186] Cheng C.P., Herfkens R.J., Taylor C.A. (2003). Comparison of abdominal aortic hemodynamics between men and women at rest and during lower limb exercise. J. Vasc. Surg..

[187] Cheng C.P., Herfkens R.J., Taylor C.A. (2003). Abdominal aortic hemodynamic conditions in healthy subjects aged 50-70 at rest and during lower limb exercise: In vivo quantification using MRI. Atherosclerosis.

[188] Malek A.M., Alper S.L., Izumo S. (1999). Hemodynamic shear stress and its role in atherosclerosis. JAMA.

[189] Koutsiaris A.G., Tachmitzi S.V., Batis N., Kotoula M.G., Karabatsas C.H., Tsironi E., Chatzoulis D.Z. (2007). Volume flow and wall shear stress quantification in the human conjunctival capillaries and post-capillary venules in vivo. Biorheology.

[190] Davies P.F. (1995). Flow-mediated endothelial mechanotransduction. Physiol. Rev..

[191] Ku D.N., Giddens D.P. (1983). Pulsatile flow in a model carotid bifurcation. Arteriosclerosis.

[192] Ku D.N., Giddens D.P., Zarins C.K., Glagov S. (1985). Pulsatile flow and atherosclerosis in the human carotid bifurcation. Positive correlation between plaque location and low oscillating shear stress. Arteriosclerosis.

[193] Nerem R.M., Harrison D.G., Taylor W.R., Alexander R.W. (1993). Hemodynamics and vascular endothelial biology. J. Cardiovasc. Pharmacol..

[194] Helmlinger G., Geiger R.V., Schreck S., Nerem R.M. (1991). Effects of pulsatile flow on cultured vascular endothelial cell morphology. J. Biomech. Eng..

[195] Chen L., Deng H., Cui H., Fang J., Zuo Z., Deng J., Li Y., Wang X., Zhao L. (2018). Inflammatory responses and inflammation-associated diseases in organs. Oncotarget.

[196] Hoesel B., Schmid J.A. (2013). The complexity of NF-κB signaling in inflammation and cancer. Mol. Cancer.

[197] Zhang T., Ma C., Zhang Z., Zhang H., Hu H. (2021). NF-κB signaling in inflammation and cancer. MedComm.

[198] Turner M.D., Nedjai B., Hurst T., Pennington D.J. (2014). Cytokines and chemokines: At the crossroads of cell signalling and inflammatory disease. Biochim. Biophys. Acta.

[199] Kany S., Vollrath J.T., Relja B. (2019). Cytokines in Inflammatory Disease. Int. J. Mol. Sci..

[200] Scioli M.G., Storti G., D’Amico F., Rodríguez Guzmán R., Centofanti F., Doldo E., Céspedes Miranda E.M., Orlandi A. (2020). Oxidative stress and new pathogenetic mechanisms in endothelial dysfunction: Potential diagnostic biomarkers and therapeutic targets. J. Clin. Med..

[201] Theofilis P., Sagris M., Oikonomou E., Antonopoulos A.S., Siasos G., Tsioufis C., Tousoulis D. (2021). Inflammatory mechanisms contributing to endothelial dysfunction. Biomedicines.

[202] Wang L., Cheng C.K., Yi M., Lui K.O., Huang Y. (2022). Targeting endothelial dysfunction and inflammation. J. Mol. Cell Cardiol..

[203] Mahler G.J., Frendl C.M., Cao Q., Butcher J.T. (2014). Effects of shear stress pattern and magnitude on mesenchymal transformation and invasion of aortic valve endothelial cells. Biotechnol. Bioeng..

[204] Liang J., Li Y., Chen L., Xia W., Wu G., Tong X., Su C., He J., Lin X., Tao J. (2019). Systemic microvascular rarefaction is correlated with dysfunction of late endothelial progenitor cells in mild hypertension: A substudy of EXCAVATION-CHN1. J. Transl. Med..

[205] Goligorsky M.S. (2010). Microvascular rarefaction: The decline and fall of blood vessels. Organogenesis.

[206] Levy B.I., Ambrosio G., Pries A.R., Struijker-Boudier H.A. (2001). Microcirculation in hypertension: A new target for treatment?. Circulation.

[207] Humphrey J.D., Schwartz M.A. (2021). Vascular mechanobiology: Homeostasis, adaptation, and disease. Annu. Rev. Biomed. Eng..

[208] Ando J., Yamamoto K. (2009). Vascular mechanobiology endothelial cell responses to fluid shear stress. Circ. J..

[209] Coleman H.A., Tare M., Parkington H.C. (2004). Endothelial potassium channels, endothelium-dependent hyperpolarization and the regulation of vascular tone in health and disease. Clin. Exp. Pharmacol. Physiol..

[210] Goto K., Ohtsubo T., Kitazono T. (2018). Endothelium-Dependent Hyperpolarization (EDH) in Hypertension: The Role of Endothelial Ion Channels. Int. J. Mol. Sci..

[211] Gerhold K.A., Schwartz M.A. (2016). Ion Channels in Endothelial Responses to Fluid Shear Stress. Physiology.

[212] Gautam M., Shen Y., Thirkill T.L., Douglas G.C., Barakat A.I. (2006). Flow-activated chloride channels in vascular endothelium: Shear stress sensitivity, desensitization dynamics, and physiological implications. J. Biol. Chem..

[213] Thakore P., Earley S. (2019). Transient Receptor Potential Channels and Endothelial Cell Calcium Signaling. Compr. Physiol..

[214] Aslam M., Gündüz D., Troidl C., Heger J., Hamm C.W., Schulz R. (2021). Purinergic Regulation of Endothelial Barrier Function. Int. J. Mol. Sci..

[215] Zheng Q., Zou Y., Teng P., Chen Z., Wu Y., Dai X., Li X., Hu Z., Wu S., Xu Y. (2022). Mechanosensitive Channel PIEZO1 Senses Shear Force to Induce KLF2/4 Expression via CaMKII/MEKK3/ERK5 Axis in Endothelial Cells. Cells.

[216] Lai A., Chen Y.C., Cox C.D., Jaworowski A., Peter K., Baratchi S. (2021). Analyzing the shear-induced sensitization of mechanosensitive ion channel Piezo-1 in human aortic endothelial cells. J. Cell Physiol..

[217] Hyman A.J., Tumova S., Beech D.J. (2017). Piezo1 Channels in Vascular Development and the Sensing of Shear Stress. Curr. Top. Membr..

[218] Friedrich E.E., Hong Z., Xiong S., Zhong M., Di A., Rehman J., Komarova Y.A., Malik A.B. (2019). Endothelial cell Piezo1 mediates pressure-induced lung vascular hyperpermeability via disruption of adherens junctions. Proc. Natl. Acad. Sci. USA.

[219] Ando J., Komatsuda T., Kamiya A. (1988). Cytoplasmic calcium response to fluid shear stress in cultured vascular endothelial cells. Vitr. Cell. Dev. Biol..

[220] Yamamoto K., Sokabe T., Ohura N., Nakatsuka H., Kamiya A., Ando J. (2003). Endogenously released ATP mediates shear stress-induced Ca^2+^ influx into pulmonary artery endothelial cells. Am. J. Physiol.-Heart Circ. Physiol..

[221] Yamamoto K., Shimizu N., Obi S., Kumagaya S., Taketani Y., Kamiya A., Ando J. (2007). Involvement of cell surface ATP synthase in flow-induced ATP release by vascular endothelial cells. Am. J. Physiol.-Heart Circ. Physiol..

[222] Chen K.-D., Li Y.-S., Kim M., Li S., Yuan S., Chien S., Shyy J.Y. (1999). Mechanotransduction in response to shear stress: Roles of receptor tyrosine kinases, integrins, and Shc. J. Biol. Chem..

[223] Gudi S.R., Clark C.B., Frangos J.A. (1996). Fluid flow rapidly activates G proteins in human endothelial cells: Involvement of G proteins in mechanochemical signal transduction. Circ. Res..

[224] Chachisvilis M., Zhang Y.-L., Frangos J.A. (2006). G protein-coupled receptors sense fluid shear stress in endothelial cells. Proc. Natl. Acad. Sci. USA.

[225] Iomini C., Tejada K., Mo W., Vaananen H., Piperno G. (2004). Primary cilia of human endothelial cells disassemble under laminar shear stress. J. Cell Biol..

[226] Nauli S.M., Kawanabe Y., Kaminski J.J., Pearce W.J., Ingber D.E., Zhou J. (2008). Endothelial cilia are fluid shear sensors that regulate calcium signaling and nitric oxide production through polycystin-1. Circulation.

[227] Wang G., Kostidis S., Tiemeier G.L., Sol W., de Vries M.R., Giera M., Carmeliet P., van den Berg B.M., Rabelink T.J. (2020). Shear Stress Regulation of Endothelial Glycocalyx Structure Is Determined by Glucobiosynthesis. Arter. Thromb. Vasc. Biol..

[228] Mochizuki S., Vink H., Hiramatsu O., Kajita T., Shigeto F., Spaan J.A., Kajiya F. (2003). Role of hyaluronic acid glycosaminoglycans in shear-induced endothelium-derived nitric oxide release. Am. J. Physiol.-Heart Circ. Physiol..

[229] Siegel G., Walter A., Kauschmann A., Malmsten M., Buddecke E. (1996). Anionic biopolymers as blood flow sensors. Biosens. Bioelectron..

[230] Schwartz M.A. (2010). Integrins and extracellular matrix in mechanotransduction. Cold Spring Harb. Perspect. Biol..

[231] Boppart M.D., Mahmassani Z.S. (2019). Integrin signaling: Linking mechanical stimulation to skeletal muscle hypertrophy. Am. J. Physiol.-Cell Physiol..

[232] Pang X., He X., Qiu Z., Zhang H., Xie R., Liu Z., Gu Y., Zhao N., Xiang Q., Cui Y. (2023). Targeting integrin pathways: Mechanisms and advances in therapy. Signal Transduct. Target. Ther..

[233] Sawada Y., Sheetz M.P. (2002). Force transduction by Triton cytoskeletons. J. Cell Biol..

[234] Katoh K., Kano Y., Ookawara S. (2008). Role of stress fibers and focal adhesions as a mediator for mechano-signal transduction in endothelial cells in situ. Vasc. Health Risk Manag..

[235] Tzima E., Del Pozo M.A., Shattil S.J., Chien S., Schwartz M.A. (2001). Activation of integrins in endothelial cells by fluid shear stress mediates Rho-dependent cytoskeletal alignment. EMBO J..

[236] Wojciak-Stothard B., Ridley A.J. (2002). Rho GTPases and the regulation of endothelial permeability. Vasc. Pharmacol..

[237] Kurazumi H., Kubo M., Ohshima M., Yamamoto Y., Takemoto Y., Suzuki R., Ikenaga S., Mikamo A., Udo K., Hamano K. (2011). The effects of mechanical stress on the growth, differentiation, and paracrine factor production of cardiac stem cells. PLoS ONE.

[238] Kendall R.T., Feghali-Bostwick C.A. (2014). Fibroblasts in fibrosis: Novel roles and mediators. Front. Pharmacol..

[239] Liu X., Liu L., Zhao J., Wang H., Li Y. (2022). Mechanotransduction regulates inflammation responses of epicardial adipocytes in cardiovascular diseases. Front. Endocrinol..

[240] John L., Ko N.L., Gokin A., Gokina N., Mandala M., Osol G. (2018). The Piezo1 cation channel mediates uterine artery shear stress mechanotransduction and vasodilation during rat pregnancy. Am. J. Physiol. Heart Circ. Physiol..

[241] Iring A., Jin Y.J., Albarran-Juarez J., Siragusa M., Wang S., Dancs P.T., Nakayama A., Tonack S., Chen M., Kunne C. (2019). Shear stress-induced endothelial adrenomedullin signaling regulates vascular tone and blood pressure. J. Clin. Investig..

[242] Roux E., Bougaran P., Dufourcq P., Couffinhal T. (2020). Fluid Shear Stress Sensing by the Endothelial Layer. Front. Physiol..

[243] Majed B.H., Khalil R.A. (2012). Molecular mechanisms regulating the vascular prostacyclin pathways and their adaptation during pregnancy and in the newborn. Pharmacol. Rev..

[244] Su J.B. (2015). Vascular endothelial dysfunction and pharmacological treatment. World J. Cardiol..

[245] Zhou J., Li Y.S., Chien S. (2014). Shear stress-initiated signaling and its regulation of endothelial function. Arter. Thromb. Vasc. Biol..

[246] Baeyens N., Bandyopadhyay C., Coon B.G., Yun S., Schwartz M.A. (2016). Endothelial fluid shear stress sensing in vascular health and disease. J. Clin. Investig..

[247] Libby P., Ridker P., Hansson G. (2009). Leducq Transatlantic Network on Atherothrombosis. Inflamm. Atheroscler. Pathophysiol. Pract. J. Am. Coll. Cardiol..

[248] Culver J.C., Dickinson M.E. (2010). The effects of hemodynamic force on embryonic development. Microcirculation.

[249] Hove J.R., Köster R.W., Forouhar A.S., Acevedo-Bolton G., Fraser S.E., Gharib M. (2003). Intracardiac fluid forces are an essential epigenetic factor for embryonic cardiogenesis. Nature.

[250] Le Noble F., Moyon D., Pardanaud L., Yuan L., Djonov V., Matthijsen R., Bréant C., Fleury V., Eichmann A. (2004). Flow regulates arterial-venous differentiation in the chick embryo yolk sac. Development.

[251] Jain R.K. (2003). Molecular regulation of vessel maturation. Nat. Med..

[252] Wang D.L., Wung B.-S., Shyy Y.-J., Lin C.-F., Chao Y.-J., Usami S., Chien S. (1995). Mechanical strain induces monocyte chemotactic protein-1 gene expression in endothelial cells: Effects of mechanical strain on monocyte adhesion to endothelial cells. Circ. Res..

[253] Virdis A. (2016). Endothelial dysfunction in obesity: Role of inflammation. High Blood Press. Cardiovasc. Prev..

[254] Gallo G., Volpe M., Savoia C. (2022). Endothelial dysfunction in hypertension: Current concepts and clinical implications. Front. Med..

[255] Han Y., Kim S.Y. (2023). Endothelial senescence in vascular diseases: Current understanding and future opportunities in senotherapeutics. Exp. Mol. Med..

[256] Brown I.A., Diederich L., Good M.E., DeLalio L.J., Murphy S.A., Cortese-Krott M.M., Hall J.L., Le T.H., Isakson B.E. (2018). Vascular smooth muscle remodeling in conductive and resistance arteries in hypertension. Arterioscler. Thromb. Vasc. Biol..

[257] Ma Z., Mao C., Jia Y., Fu Y., Kong W. (2020). Extracellular matrix dynamics in vascular remodeling. Am. J. Physiol.-Cell Physiol..

[258] Hagiwara M., Koh I. (2020). Engineering approaches to control and design the in vitro environment towards the reconstruction of organs. Dev. Growth Differ..

[259] Gordon E., Schimmel L., Frye M. (2020). The importance of mechanical forces for in vitro endothelial cell biology. Front. Physiol..

[260] Krishnan R., Park J.-A., Seow C.Y., Lee P.V., Stewart A.G. (2016). Cellular biomechanics in drug screening and evaluation: Mechanopharmacology. Trends Pharmacol. Sci..

[261] Yang L., Pijuan-Galito S., Rho H.S., Vasilevich A.S., Eren A.D., Ge L., Habibovic P., Alexander M.R., de Boer J., Carlier A. (2021). High-throughput methods in the discovery and study of biomaterials and materiobiology. Chem. Rev..

[262] Doran S., Arif M., Lam S., Bayraktar A., Turkez H., Uhlen M., Boren J., Mardinoglu A. (2021). Multi-omics approaches for revealing the complexity of cardiovascular disease. Brief. Bioinform..

