# Supplementary material for: Effects of Mechanical Stress on Endothelial Cells In Situ and In Vitro
Source: Int J Mol Sci. 2023 Nov 20;24(22):16518. doi: 10.3390/ijms242216518 (PMC10671803; doi:10.3390/ijms242216518)
Supplement: Supplementary file 1 [file ijms-24-16518-s001.zip › ijms-2652338-supplementary/Supplemental material explanation.pdf]

Supplemental video1 (S1)
Simulation of the velocity streamline of coarctation zone of abdominal aorta.

Supplemental video2 (S2)
Simulation of the velocity contour plotting in iliac artery with pulsatile flow.

Supplemental video3 (S3)
Simulation of the velocity streamline in plane in iliac artery with pulsatile flow.

Suplemental video4 (S4)
Simulation of the wall shear stress distribution in iliac artery with pulsatile flow.
